# Cell-free DNA donor fraction analysis in pediatric and adult heart transplant patients by multiplexed allele-specific quantitative PCR: Validation of a rapid and highly sensitive clinical test for stratification of rejection probability

**DOI:** 10.1371/journal.pone.0227385

**Published:** 2020-01-13

**Authors:** Paula E. North, Emily Ziegler, Donna K. Mahnke, Karl D. Stamm, Angela Thomm, Paul Daft, Mary Goetsch, Huan ling Liang, Maria Angeles Baker, Adam Vepraskas, Chris Rosenau, Mahua Dasgupta, Pippa Simpson, Michael E. Mitchell, Aoy Tomita-Mitchell

**Affiliations:** 1 Department of Pathology, Medical College of Wisconsin, Milwaukee, Wisconsin, United States of America; 2 Childrens Hospital of Wisconsin, Milwaukee, Wisconsin, United States of America; 3 TAI Diagnostics, Inc., Wauwatosa, Wisconsin, United States of America; 4 Department of Surgery, Medical College of Wisconsin, Milwaukee, Wisconsin, United States of America; 5 Department of Pediatrics, Medical College of Wisconsin, Milwaukee, Wisconsin, United States of America; Thomas Jefferson University, UNITED STATES

## Abstract

Lifelong noninvasive rejection monitoring in heart transplant patients is a critical clinical need historically poorly met in adults and unavailable for children and infants. Cell-free DNA (cfDNA) donor-specific fraction (DF), a direct marker of selective donor organ injury, is a promising analytical target. Methodological differences in sample processing and DF determination profoundly affect quality and sensitivity of cfDNA analyses, requiring specialized optimization for low cfDNA levels typical of transplant patients. Using next-generation sequencing, we previously correlated elevated DF with acute cellular and antibody-mediated rejection (ACR and AMR) in pediatric and adult heart transplant patients. However, next-generation sequencing is limited by cost, TAT, and sensitivity, leading us to clinically validate a rapid, highly sensitive, quantitative genotyping test, myTAI_HEART_^®^, addressing these limitations. To assure pre-analytical quality and consider interrelated cfDNA measures, plasma preparation was optimized and total cfDNA (TCF) concentration, DNA fragmentation, and DF quantification were validated in parallel for integration into myTAI_HEART_ reporting. Analytical validations employed individual and reconstructed mixtures of human blood-derived genomic DNA (gDNA), cfDNA, and gDNA sheared to apoptotic length. Precision, linearity, and limits of blank/detection/quantification were established for TCF concentration, DNA fragmentation ratio, and DF determinations. For DF, multiplexed high-fidelity amplification followed by quantitative genotyping of 94 SNP targets was applied to 1168 samples to evaluate donor options in staged simulations, demonstrating DF call equivalency with/without donor genotype. Clinical validation studies using 158 matched endomyocardial biopsy-plasma pairs from 76 pediatric and adult heart transplant recipients selected a DF cutoff (0.32%) producing 100% NPV for ≥2R ACR. This supports the assay’s conservative intended use of stratifying low versus increased probability of ≥2R ACR. myTAI_HEART_ is clinically validated for heart transplant recipients ≥2 months old and ≥8 days post-transplant, expanding opportunity for noninvasive transplant rejection assessment to infants and children and to all recipients >1 week post-transplant.

## Introduction

Noninvasive risk assessment for rejection in heart transplant recipients, both adult and pediatric, is an imperative and urgent clinical need. Organ-transplant patients require life-long immunosuppression that must be controlled carefully to balance risk of allograft rejection and loss with equally life-threatening immunosuppression-induced risks of infection, cancer, and other maladies. In heart transplant patients, this balance traditionally has been monitored through a multitude of diagnostic modalities. These include assessments of clinical symptomology, viral loads and other microbiological indicators, immunosuppressive drug and procalcitonin blood levels [1], echocardiography [2], cardiac magnetic resonance imaging [3], noninvasive measurements of levels of circulating donor-specific antibodies and cardiac-derived proteins such as troponin [4] and B-type natriuretic peptide hormone (NT-proBNP) [5,6,7], and surveillance or symptom-prompted application of endomyocardial biopsy (EMB) with or without concurrent coronary angiography. EMB is the historical and still current gold standard for assessment of cardiac allograft acute cellular and antibody-mediated rejection (ACR and AMR) due to its direct histological visualization of myocardial and/or intravascular inflammatory infiltration and cellular injury. It routinely is combined with immunohistochemical or immunofluorescent detection of C4d capillary deposition as a surrogate for classical complement activation that assists in evaluation for AMR [8–11]. Despite its strengths, utility of EMB is limited by requirement for adequate vascular access, significant and even life-threatening risks imposed by intra-cardiac sampling, need for repeated anesthesia, diagnostic sampling error due to biopsy site restriction and need for multiple samples to increase sensitivity due to the inherently patchy histological distribution of myocardial inflammatory infiltrates in rejection [12–19]. EMB is also hampered by alarmingly low inter-observer (pathologist) concordance in assignment of rejection grade despite modifications in grading criteria [20–23]. In addition, the incidence of “biopsy negative” rejection evidenced by hemodynamic compromise without demonstrable myocardial inflammation remains at approximately 20% [23–26]. It is thus a problematic gold standard, and some centers seek to reduce incidence of its use, particularly in infants and children, but also in adults after the first year post-transplant [27]. To do this with improved confidence, noninvasive, relatively inexpensive testing alternatives with high negative predictive value (NPV) for significant rejection are needed to provide adequate, even increased, frequency of monitoring to detect rejection before it becomes clinically evident [28,29].

Two early clinical offerings for noninvasive monitoring of heart transplant rejection included the Heartsbreath^™^ test (Menssana Research, Inc.) and the AlloMap^®^ test (CareDx, Brisbane, CA). Heartsbreath^™^ is a test for biomarkers of oxidative stress in breath and received FDA approval in 2004 through humanitarian device exception. That approval was based on a single, non-randomized three-year multicenter National Heart Lung and Blood Institute-sponsored study of 539 heart transplantation patients in which breath was analyzed by gas chromatography/mass spectroscopy for alkanes and monomethyl alkanes and correlated with histological grading of subsequent EMB [30,31]. This test has extremely limited indication for use as an aid in the diagnosis of grade 3 heart transplant rejection in patients who have received heart transplants within the preceding year and have had endomyocardial biopsy within the previous month; results have not been corroborated independently and additional publications have not accrued. AlloMap is based on RNA-based leukocyte gene-expression profiling (GEP) to stratify risk of ACR in heart transplant patients and is reported as a combined AlloMap Score ranging from 0 to 40 for a subset of leukocyte inflammatory responses without provision of a specific cut-off level for a positive test. AlloMap scoring has been positively associated in some studies with risk of moderate to severe ACR in adult heart transplant patients [23,32]. AlloMap GEP scoring positive predictive value (PPV) for ISHLT 2004 ACR grades ≥2R is low (<5%), whereas NPV is near 100% [23], making it primarily applicable as an alternative for EMB in stable patients within low-risk populations more than 6 months post-transplant [33]. Transplant vasculopathy has also been associated with increased AlloMap score [34]. AlloMap analysis was not designed to detect AMR and is not approved for clinical use in children under 15 years of age or within < 55 days of transplantation. AlloMap score does not correlate strongly with ACR in pediatric heart transplant recipients, tends to trend upward over time after transplantation independently of graft function [32,35], and is directly influenced by widely utilized therapeutic suppressors of allograft rejection such as sirolimus, tacrolimus, and corticosteroids [36].

A more widely applicable and direct biomarker for risk stratification of transplant rejection, the donor-specific fraction (DF) of recipient plasma cell free DNA (cfDNA), has also been explored [37–39] and is supported by recent clinical success of conceptually similar diagnostic application of chimeric cfDNA analysis in molecular maternal-fetal medicine [40–42] and cancer diagnostics [43–47]. Plasma cfDNA in healthy individuals, first described in the 1940’s [48], is largely the result of fragmented nuclear DNA release during normal apoptotic cellular turnover, displaying a ladder-like size distribution peaking at ~167bp in length corresponding to multiples of nuclease resistant chromatosomes which consist of complexes of cfDNA and nucleosomes [40,49]. Mitochondrial cfDNA is also present in normal plasma, predominating over nuclear-derived cfDNA in units of copy number/ml but approximately 4 times less in ng/ml [50,51]. A smaller, but biologically impactful, component of DNA is released from living cells under metabolic control by processes including exocytosis and NETosis (neutrophil extracellular traps) [52,53]. The low level of DNA release into the circulation seen in good health becomes elevated in response to induced cellular injury in various pathologic states including sepsis and severe infections, trauma, ischemic injury, autoimmune disease, and cancer [54–59]. Transient non-pathologic cfDNA elevations also occur after intense or prolonged exercise [60], but rapidly return to baseline upon recovery, consistent with the short half-life of cfDNA fragments in plasma, which generally ranges from 4–30 min [61–63]. Despite complexity of cfDNA origins in mammalian biology and need for consideration of contributing clinical conditions, the preponderant generation of donor-specific and recipient-specific cfDNA from cellular apoptosis in transplant patients and its relatively short plasma half-life make cfDNA DF an elegant and dependable temporal indicator of ongoing selective injury to the donor organ.

Early iterations of cfDNA-based assays for determination of plasma DF in transplant recipients were challenged by the very low levels of cfDNA in plasma of healthy transplant recipients, which closely overlap those of un-transplanted healthy controls, much lower than levels typically observed in cancer patients and during pregnancy. Accordingly, some have targeted known donor-recipient discriminative loci, such as Y chromosome-specific sites (*e*.*g*., SRY) in sex-mismatched donor-recipient pairs, of clearly limited application, and HLA genes, limited by poor informativity of target sequences in some recipient-donor pairs [37,38,61,64,65]. More recently, advanced technologies such as multiplexed high-fidelity amplification combined with allele-specific real-time quantitative PCR [66] and newer versions of next generation sequencing (NGS) [39,67] have been leveraged. These new methods improve sensitivity by interrogating a large multiplicity of highly informative single nucleotide polymorphism (SNP) sites, empowering prospective clinical studies that correlate cfDNA DF with biopsy-documented transplant rejection grade in well-defined patient populations of heart [39,66,68,69], kidney [70–72], liver [73] and lung [67] transplant recipients. The foundational tenants of DF chimeric analysis are generally applicable to all organ transplantation categories, and bioinformatic advancements using these newer technologies no longer require donor sample availability to determine DF in unrelated and most related donor-recipient pairs [66,74].

Methodological differences in DF measurement define the performance characteristics, speed, cost, and practicality of clinical application and contribute importantly to its clinical utility. Digital PCR has been explored to improve sensitivity of DF determination and lower cost, but is hampered by limited multiplexing capacity [75]. Using NGS, we previously demonstrated a strong positive correlation between elevated DF and both ACR and AMR in pediatric and adult heart transplant patients [39]. However, standard targeted NGS is significantly limited by its cost, turnaround time (TAT), and level of sensitivity imposed by background noise, leading us to develop a rapid, highly sensitive, cost-effective multiplexed allele-specific PCR test, termed myTAI_HEART_^®^ to address the limitations of NGS while also eliminating need for donor genotyping. myTAI_HEART_ is capable of early detection of mild ACR (ISHLT 1R) in addition to higher grade ACR (ISHLT 2R and 3R), AMR, and graft vasculopathy. We herein report results of analytical and clinical validation studies performed to support clinical diagnostic use of this non-invasive test for quantitative determination of total cfDNA (TCF) concentration and cfDNA DF with subsequent stratification of risk of moderate to severe ACR in adult and pediatric heart transplant patients 2 months of age or older and 8 or more days post-transplant. Donor genotyping is not required. This provides an unmet clinical need for virtually all heart transplant patients in the most critical period for rejection risk as well as throughout life.

## Materials and methods

### Overview of assay design and testing workflow

**Fig 1:**
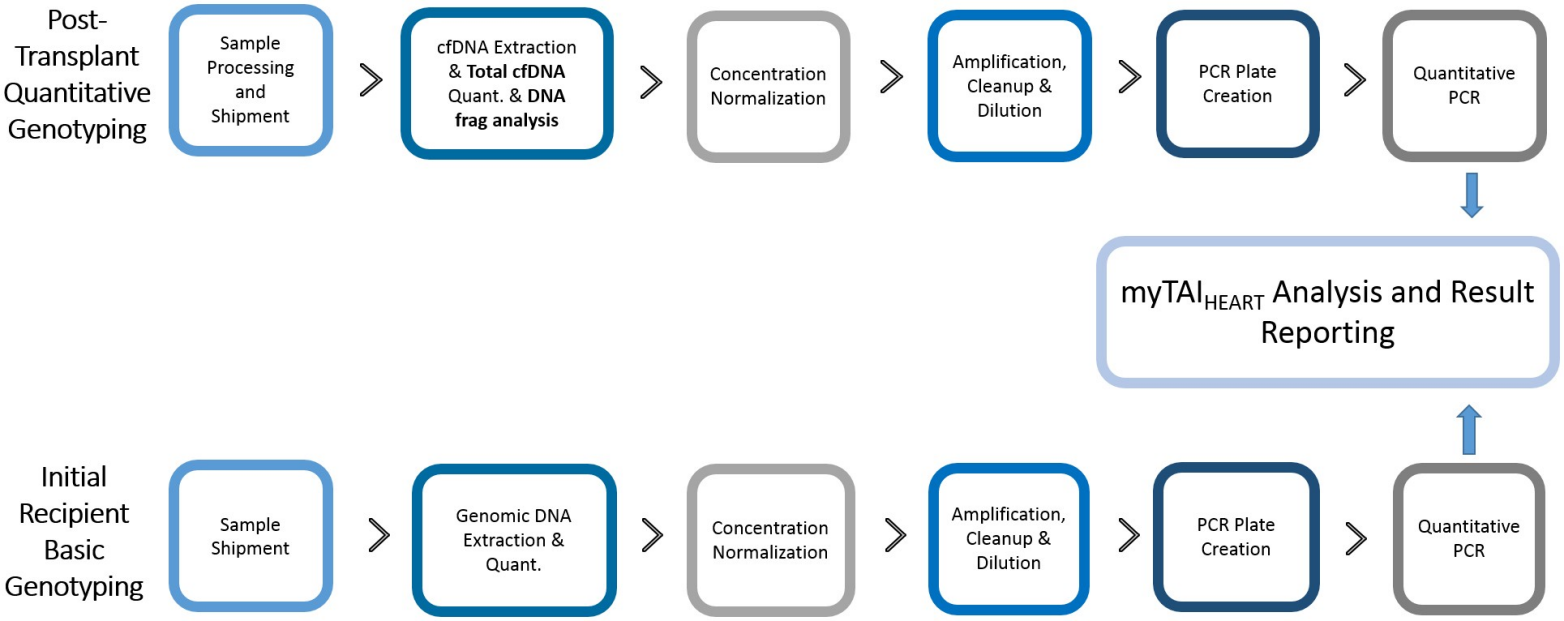
myTAI_HEART_ clinical testing workflow schematic.

The myTAI_HEART_ test uses multiplexed, high-fidelity amplification followed by allele-specific qPCR of a panel of 94 highly informative bi-allelic single nucleotide polymorphisms (SNPs) and two controls in a heart transplant recipient’s plasma, thereby distinguishing “donor specific” cfDNA originating from the engrafted heart from “self-specific” cfDNA originating from the recipient’s own native cells. Fig 1 depicts a simplified schematic of the overall workflow from sample receipt to final reporting.

As a one-time clinical requirement for initiation of myTAI_HEART_ testing on any given transplant recipient, a sample of recipient whole blood, either pre- or post-transplant, is collected in an EDTA tube and without further processing sent to TAI Diagnostics for basic genotyping (bGT) of the recipient’s genomic (leukocyte) DNA (gDNA) at the 94 SNPs of interest. Plasma cfDNA DF for that recipient at clinically indicated time points post-transplant is then determined in subsequent blood samples by multiplexed, high-fidelity amplification and allele-specific quantitative genotyping (qGT) of those same targets. The blood samples for post-transplant qGT are collected as whole blood in a K_2_EDTA plasma gel separator tube (BD Vacutainer^®^ PPT^™^ Plasma Preparation Tube) and require subjection to two brief low speed centrifugation steps at the collection site within 2 hours of phlebotomy. This allows quick and effective separation of the plasma (cfDNA) phase from contaminating leukocytes and erythrocytes prior to freezing and shipping overnight to TAI Diagnostics for subsequent qGT to determine TCF concentration (ng/ml) and cfDNA DF (%). qGT is performed each time a plasma sample is submitted for myTAI_HEART_ testing, producing a unique longitudinal post-transplant time point for determination of plasma TCF concentration and DF. DF provides a point estimation of selective injury to donor-specific cells of the transplanted heart that is used to categorize the patient’s probability of transplant rejection.

Upon receipt in the TAI Clinical Reference Laboratory, whole blood and frozen plasma samples are extracted and processed through bGT and/or qGT workflows as appropriate. Prior to entering these workflows, gDNA from the recipient whole blood sample (for bGT) is extracted and quantitated by UV-spectroscopy. cfDNA is extracted from the plasma sample (for qGT), quantified by reference gene quantitative PCR = RNase P qPCR) and subjected to a proprietary, internally controlled myTAI_HEART_ DNA fragmentation test. The myTAI_HEART_ DNA Fragmentation Assay is based on fragment length-dependent real time qPCR amplification of multi-copy *Alu* gene sequence and is used as a quality control step to rule out significant contribution to the cfDNA pool by leukocyte lysis or contamination during sample collection and plasma separation that may necessitate specimen rejection. The one-time bGT result is used for interrogating qGT findings to generate the cfDNA DF using the proprietary myTAI_HEART_ software.

For each incidence of clinical myTAI_HEART_ testing, a signed test report is generated resulting plasma TCF in ng/ml and DF (%). Based on the DF result, an interpretation is further assigned, according to validated reference ranges (see *Clinical Validation* below), for low versus increased probability of moderate or severe ACR. Additional report content includes test performance characteristics, intended use, historical DF results for the patient, warnings and limitations, and optional comments from the Medical Director. All positive results for increased risk of rejection are critical results and directly communicated to the ordering physician or other responsible health care provider. Copies of the final report are sent to the referring laboratory and ordering physician by secure fax and or email as specified on the test requisition, followed by hard copy. The entire workflow of this Laboratory Developed Test (LDT) was documented formally and locked prior to performance of clinical analytical validation studies described herein in the Clinical Laboratory Improvement Act (CLIA) and College of American Pathologists (CAP)-accredited TAI Diagnostics Clinical Reference Laboratory.

### Target selection and primer design

Targets were chosen from among SNPs exhibiting variation across the entire range of known human genetic diversity in order to ensure that a single myTAI_HEART_ genotyping panel would function well without availability of the donor genotype and would be highly informative for a broad range of transplant recipients and donors with diverse ancestral and ethnic genetic backgrounds. These backgrounds included all major human supergroups of which seven were defined by gnomAD [76,77] and five were defined by the 1000 Genomes Project Consortium, 2015 [78]. The myTAI_HEART_ panel was designed initially by filtering from over 240 million known variant, strictly biallelic, sites for minor allele frequency cutoffs of > 25% in each subpopulation of the 1000 Genomes, also requiring that they be greater than 1000 base pair (bp) apart to minimize linkage. Potentially problematic genomic regions in the initially selected group were removed prior to final SNP selection by excluding syndromic regions likely to be abnormal in heart transplant patients (such as trisomy 21 and CNVs associated with disease) [79], low complexity regions such as centromeres and telomeres, and regions of high GC content or frequent STR occurrence. All candidate SNP sites were cross-referenced to both ClinVar and OMIM databases [80,81], allowing exclusion of any SNPs with known disease association. Regions +/- 35 bp around each candidate SNP define windows fitting the fragment size limit associated with cfDNA. These were screened to confirm absence of lower frequency variation (SNP and indels) which would influence primer binding.

Multiple 20–26 bp primer pairs within each selected SNP window were designed to amplify targets. To ensure specificity, all candidate primers were queried with BLAST to Human Reference Genome GCRh37 to check for possible cross amplification of non-pairing primers to non-target sites in the human genome. Multiplex amplification was evaluated *in silico* to select compatible candidate pools. A pool of 400 candidate primer pairs [82] was chosen and tested for multiplex compatibility using a common melting temperature and reagents. Compatible targets were evaluated further for amplification efficiency and DF specificity, with the 94 members of the final myTAI_HEART_ panel selection representing the upper quartile of this pool. Median lengths of final library and allele specific primers were 42 bp and 21bp, respectively.

### Manufacture of reference materials

**Fig 2:**
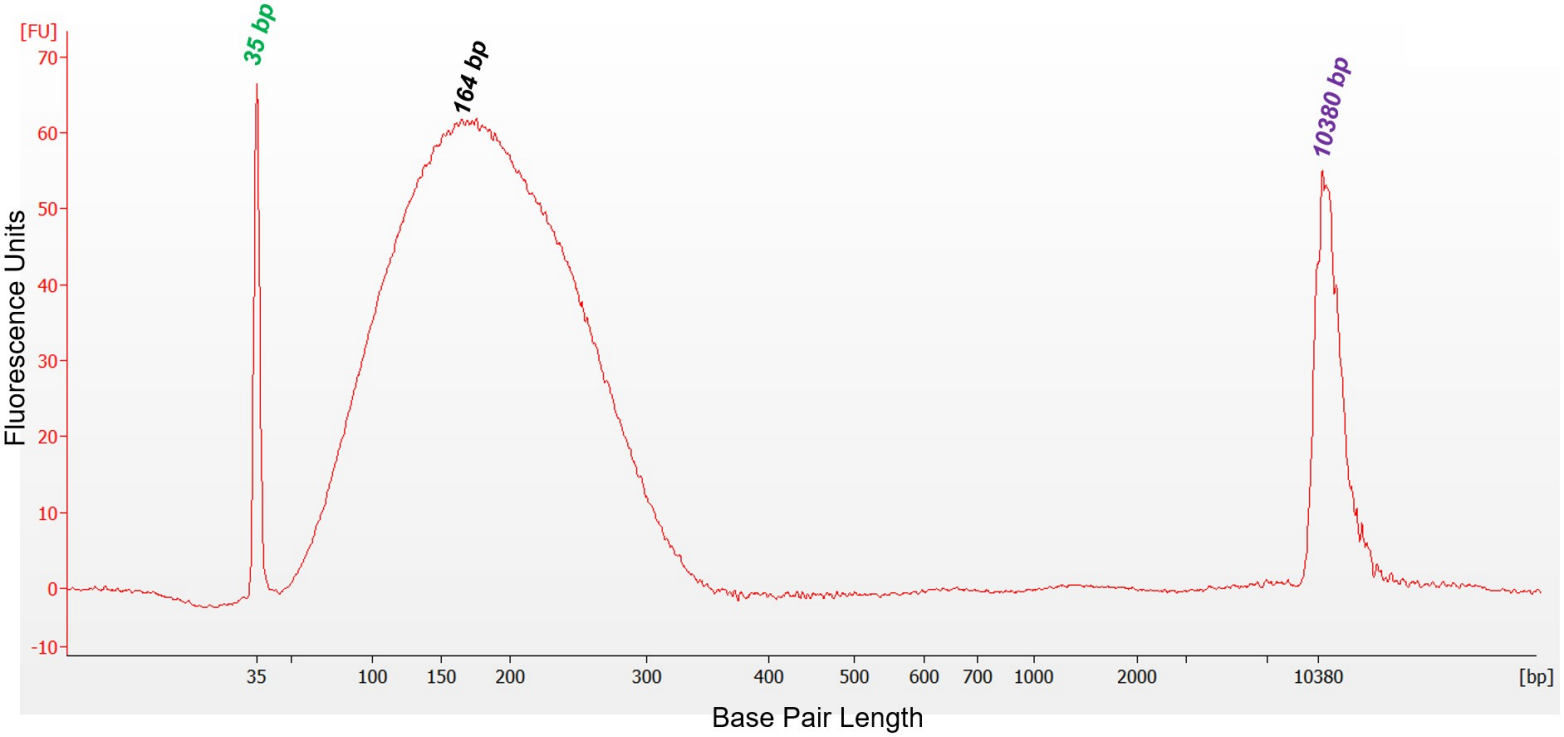
Electropherogram image of sheared gDNA, simulating cfDNA. An Agilent 2100 Bioanalyzer instrument and high sensitivity DNA Kit were used to demonstrate the 164 bp peak corresponding to the median distribution of gDNA sheared by ultrasonication to the size range of cfDNA of apoptotic origin. FU, fluorescence units; bp, base pairs. Peaks at 35 and 10380 bp represent lower and upper internal kit standards.

The validation studies reported here were dependent upon large volumes of input plasma to test all variables in an appropriate number of replicates while also providing the appropriate range of cfDNA concentration and cfDNA DF. Accordingly, contrived reference materials consisting of specified combinations of human plasma samples, human cfDNA and gDNA isolates, and sheared human gDNA preparations were developed and manufactured at TAI Diagnostics to support validation study needs, including provision of controls. Unless otherwise stated, all plasma samples were isolated from whole blood sourced from a commercial vendor. Plasma was separated from whole blood by centrifuging at 1400 x g for 10 minutes, removed and centrifuged a second time at 1400 x g for 10 minutes, followed by a third centrifugation at 15,000 x g for 10 minutes. Aliquots of the plasma and the buffy coat preparations were frozen at -80°C until needed. For use in validation studies for TCF quantification and for the myTAI_HEART_ DNA Fragmentation Assay, plasma was spiked with short fragments of DNA obtained by Covaris ME220 (Woodburn, MA) focused ultrasonication (“shearing”) of gDNA from the paired cellular component (buffy coat) to a size distribution primarily in the range of 130–180 bp, approximating that of cfDNA. Resultant fragment lengths were evaluated on an Agilent 2100 Bioanalyzer (Santa Clara, CA) with a high sensitivity DNA chip to confirm production of the targeted range as determined by base pair size of maximum fluorescence values (Fig 2). Prior to final manufacture using these contrived materials for validation studies, feasibility studies were performed to demonstrate they produced materials of targeted cfDNA concentrations and donor fractions when tested through the myTAI_HEART_ workflow (S1 Fig). For qGT validation studies, gDNA was isolated in bulk and quantified by NanoDrop One UV-spectroscopy. The quantified DNA was used, without further manipulation, to make precise reconstructions consisting of DNA from two individuals mixed together at a broad range of specified concentrations to simulate samples isolated from transplant patients containing both recipient and donor DNA.

### Patient blood sample collection and plasma separation

Standardized sample collection and processing protocols that minimize leukocyte lysis and produce plasma free of leukocyte contamination are important for cfDNA analyses of all types. This is particularly important for determinations of TCF concentration and DF that accurately reflect *in vivo* levels in transplant recipient blood samples, given the very low levels of TCF typically present in these patients and the low DF’s (often < 1%) associated with increased probability of rejection. The TAI Protocol for cfDNA sample handling and processing requires separation of plasma from leukocytes with two low speed spins performed within 2 hours of collection, extended to 4 hrs in Streck BCT tubes (Streck, Omaha, NE), whether for clinical trial or clinical diagnostic use. One hundred and fifty eight biopsy-associated heart transplant patient samples collected at Froedtert Lutheran Memorial Hospital (FLMH) and Children’s Hospital of Wisconsin (CHW) in Milwaukee were used in clinical validation of the myTAI_HEART_ DF test (see *Clinical Validation Design* below). Whole blood was drawn into 10 ml Cell-Free DNA BCT^®^ tubes (Streck, Omaha, NE) tubes at FLMH and CHW, hand-walked to the adjacent Children’s Research Institute (CRI) Nucleic Acid Extraction Lab from centrifugation to separate plasma (by two 1,400 x g spins) prior to freezing at -80°C for delayed extraction. DNA Based Transplant Rejection Test (DTRT) prospective blinded multicenter study samples (NIH/NHLBI, ClinicalTrials.gov Identifier: NCT02109575, CHW Institutional Review Board, CHW 10/83, GC 111,CTSI 906) were used to establish the TCF “normal” reference range for the myTAI_HEART_ test in healthy, asymptomatic heart transplant patients. These samples were also collected in 10 ml Streck BCT tubes and rapidly processed per protocol prior to submission to TAI Diagnostics for analysis (see *Clinical Validation Design* below). For myTAI_HEART_ clinical diagnostic testing, the TAI protocol requests that samples shipped distantly to TAI be collected in 8.5 ml BD Vacutainer^®^ PPT^™^ Plasma Preparation Tubes (BD Biosciences, San Jose, CA) then subjected to two brief low speed (1100xg) centrifugation steps before freezing of purified plasma for shipment overnight on dry ice to TAI Diagnostics. The PPT processing procedure was formally beta-tested in two hospital clinical laboratories to collect feedback from laboratory staff regarding ease of integration into standard laboratory workflows.

For one-time basic genotyping (bGT) of each recipient, which targets the overwhelming abundance of leukocyte genomic nuclear (and mitochondrial) DNA in whole blood, a simple K2-EDTA (purple top) whole blood collection without centrifugation was sent to TAI prior to or coincident with the first myTAI_HEART_ test submission according to standard clinical laboratory procedures. Shipment frozen or on ice was considered acceptable, but not considered superior to standard ambient temperature shipment.

### Quality control to detect leukocyte contamination/lysis in patient samples

**Fig 3:**
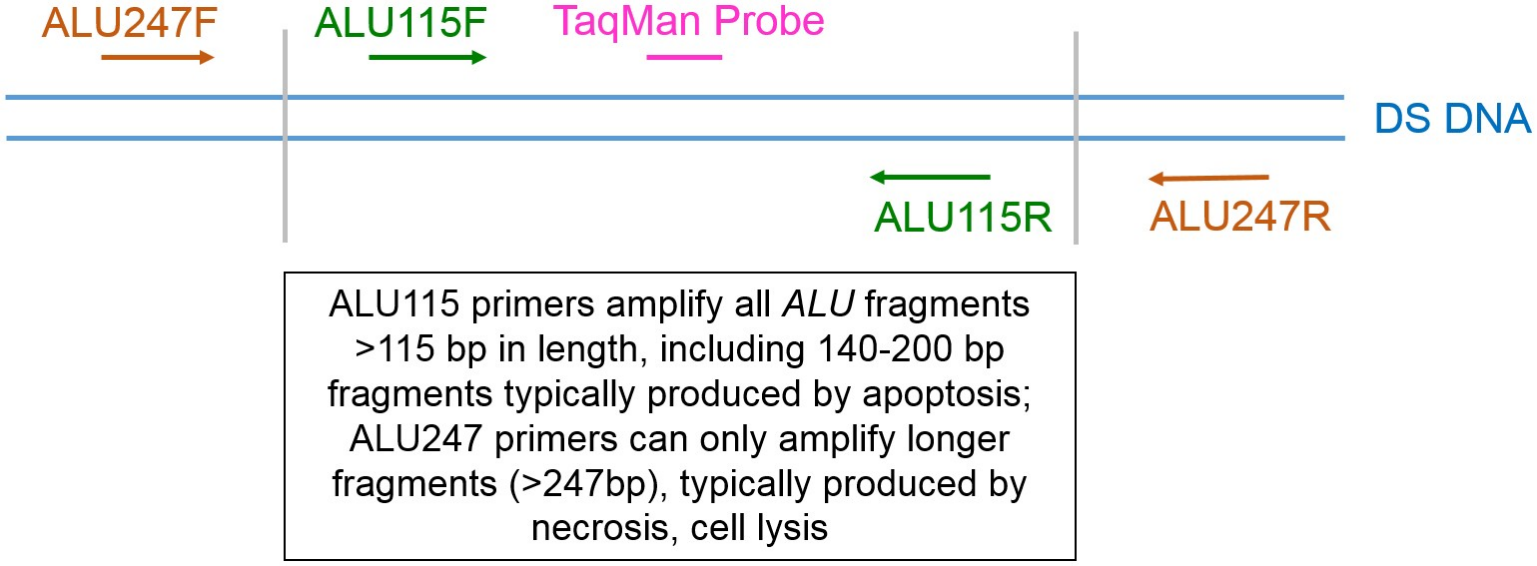
*Alu* 115bp (ALU115) and 247bp (ALU247) PCR primer designs. Forward and reverse primers of ALU115 are indicated by green text, ALU247 primers by orange text. Brackets indicate the size of fragments (140–200 bp) generated by enzymatic apoptotic cleavage as compared to the total length of the *Alu* element. ALU115 primers amplify apoptotic and longer DNA fragments, while ALU247 primers only amplify sequences longer than apoptotic DNA.

TAI sample processing protocols were designed to prevent significant leukocyte lysis and/or contamination that could reasonably dilute the DF of cfDNA through contamination by recipient-specific gDNA after sample collection. As a quality control measure, a sensitive DNA fragmentation assay based upon analysis of a multi-copy gene was developed to identify, prior to further cfDNA analysis, if unintended gDNA contamination did occur due to unrecognized sample preparation deviations. The myTAI_HEART_ DNA Fragmentation Assay is a proprietary modification of the method of Utemani et al [83] and uses primers specific for different lengths of the *Alu* multi-copy gene consensus sequence, with a shared TaqMan probe for both short and long amplicons (ALU115 and ALU247, respectively). *Alu* elements are short stretches of DNA originally characterized by the action of the Arthrobacter luteus (*Alu*) restriction endonuclease. *Alu* sequences are classified as short interspersed nucleotide elements, approximately 300 nucleotides in length, and account for more than 10% of the human genome at a copy number of about 1.4 million per genome [84,85,86]. This high copy number makes them advantageous targets for highly sensitive detection and DNA fragmentation analysis of low template populations such as circulating cfDNA in organ transplant patients. The ALU115 primer pair (Fig 3) utilized by the myTAI_HEART_ DNA Fragmentation Assay produces amplification product from *Alu* fragments of almost all lengths, including the short fragments of a modal size of about 166 bp (140–200 bp) characteristically derived from cellular apoptosis [53,87], as well as all longer *Alu* fragments, essentially the entire cfDNA complement. In contrast, only those longer fragments derived from non-apoptotic cellular death mechanisms, such as those occurring from *ex vivo* lysis of leukocytes during whole blood sample processing, are detected by amplification of a 247 bp fragment of the *Alu* sequence (Fig 3). The ratio of product from the ALU247 amplification to product from the ALU115 amplification increases as contribution by post-collection leukocyte lysis to the cfDNA pool increases. Because the annealing site of ALU115 is nested within the ALU247 annealing site, the qPCR ratio would theoretically be 1.0 when template DNA is not fragmented and 0.0 when all template DNA is truncated into fragments smaller than 247 bp. Because the ALU115 primers can amplify most fractions of circulating DNA, the ALU-qPCR result obtained with ALU115 primers in plasma samples effectively represents the absolute amount of cfDNA. The *Alu* ratio (247 bp:115 bp) provides a useful tool to detect levels of leukocyte lysis which might produce a false negative result for increased probability of rejection in samples evaluated for DF.

The myTAI_HEART_ DNA Fragmentation Assay was performed on cfDNA extract as part of the overall myTAI_HEART_ workflow after quantification of extract TCF concentration by RNase P qPCR. Input was 50 pg, run in triplicate for both *Alu* fragment length amplifications against a five-point human gDNA standard curve. ALU115 and ALU247 amplifications were performed individually for each primer pair on a Roche Lightcycler 480 (LC280) using a shared proprietary TaqMan^™^ probe. The Lightcycler software was used to calculate a standard curve for the run by plotting the known DNA concentration of each standard dilution on the x-axis and the mean crossing point (Cp) value for those dilutions on the y-axis, also calculating the slope and amplification efficiency for each run. TCF concentrations of patient samples were individually determined by the Lightcycler software for the ALU115 and ALU247 amplifications using the calculated standard curve equation and the mean Cp as input. Results generated by the LC480 Abs Quant/2nd Derivative Max algorithm captured in a report were used to determine *Alu* ratio by dividing the ALU247 concentration by the ALU115 concentration. That ratio provides a quality indicator of potentially significant leukocyte lysis/contamination of the patient sample that could potentially influence DF results and/or cause specimen rejection (see Results, *Clinical Validation*). Analytical quality metrics developed to ensure validity of a myTAI_HEART_ DNA Fragmentation Assay run included required ranges for ALU115 and ALU247 amplification efficiency, standard deviations of standard curve points, quantifications in pg/μl of low, medium, and high ALU115 and ALU247 controls, fragment ratios of specified standards, no template control (NTC) mean Cp, and specified standard Cp. For detailed analytical validation data, see Analytical Validation Methods below.

### Genomic and cfDNA extraction

cfDNA for determination of DF by qGT was extracted from 4.0 ml aliquots of patient plasma or contrived reference samples manufactured at TAI Diagnostics as described above. The extraction was performed in a standardized, automated fashion using a high throughput TECAN liquid handling platform to minimize inter-sample variability in extraction efficiency. Proprietary chemistry and scripts were employed to optimize extraction efficiency appropriate to the range of DNA fragment lengths typical of patient cfDNA mixtures. TCF concentration in extracts of plasma and contrived reference samples was determined by RNase P qPCR (TaqMan^®^ Copy Number Reference Assay RNase P, Applied Biosystems, Foster City, CA). gDNA for bGT analysis was extracted from buffy coat of whole blood collected in a standard K2-EDTA tube either manually using a Qiagen kit or in an automated fashion using the TECAN. The concentrations and A260/A280 ratios of gDNA extracts were determined by NanoDrop^™^ One (ThermoFisher Scientific, Waltham, MA) spectroscopy.

### Basic genotyping (bGT)

For each heart transplant recipient represented in the validation study set and for each volunteer blood donor providing samples for manufacture of validation reference materials, one-time basic genotyping (bGT) of gDNA extracted from a blood sample was performed at each of the 94 highly informative target allelic sites (and 2 control sites) prior to qGT. Additionally, donor whole blood samples were available for separate genotyping of donors for 158 recipients providing the matched endomyocardial biopsy—blood sample pairs used in the clinical validation study. This provided opportunity to validate the evolved “without donor genotype” version of the myTAI_HEART_ test by direct comparison to results obtained on the same patients using the original “with donor genotype” version of the test, the two versions differing in final algorithmic interrogation of the post-transplant qGT results to determine DF.

Samples for bGT entered the workflow by input of 15 ng of extracted gDNA into a multiplexed high-fidelity library amplification reaction containing 96 library primer pairs (for 94 highly informative allelic targets and 2 controls), each amplifying a region including one of the myTAI_HEART_ target sites. Heterozygous (HET), homozygous reference (HOM REF) and homozygous variant (HOM VAR) calibrators were pre-prepared from pooled mixtures of sequence-verified, double-stranded DNA linear constructs and representing each of the myTAI_HEART_ target alleles. Accompanied by a negative template control (NTC), calibrators were amplified using Q5 Hot Start High-Fidelity DNA Polymerase (New England BioLabs, Ipswich, MA) in each multiplexed library run alongside patient/validation samples on Eppendorf Mastercyclers^®^. Subsequent enzymatic cleanup (ExoSAP-IT™, Applied Biosystems, Foster City, CA) was employed to remove excess primers and unincorporated deoxynucleotide triphosphates (dNTPs), preventing interference with downstream amplification steps. Adequacy of library amplification was monitored by post-amplification automated microfluidic electrophoresis using an Agilent 2100 Bioanalyzer (Agilent Technologies, Santa Clara, CA). Passing pre-amplification library mixtures were advanced into the genotyping phase of bGT workflow, there subjected alongside HET, HOM REF, HOM VAR calibrators and NTC to robotically-controlled real-time Roche LC480 PCR amplification and product detection using AptaTaq Genotyping Master Mix (Roche, Indianapolis, IN) and the proprietary myTAI_HEART_ primers and probes (see Target Selection and Primer Design). Post-amplification data was analyzed by the myTAI_HEART_ software to provide genotype calls and associated quality metrics.

Sample quality control (QC) measures applied to ensure accurate bGT results included requirement for ≥ 0.5 mL volume of whole blood, shipment frozen or according to sender’s standard procedure, time from sample receipt to extraction ≤ 3 days, gDNA quantity ≥ 15 ng, and quality (A260/A280 ≥ 1.7 and ≤ 1.99, as determined by NanoDrop One UV spectroscopy). Genotyping QC acceptance criteria included a valid run with a minimum of 89% (84/94) of targets with passing calibrator data, and a valid sample with a specified minimum percentage of genotype calls and a specified maximum of heterozygous genotype calls.

### Quantitative genotyping (qGT): Multiplexed, high fidelity amplification followed by allele-specific qPCR

DF was determined by multiplexed, high-fidelity amplification followed by allele-specific qPCR of 94 SNP targets and 2 control targets also targeted by one-time bGT of the recipient’s native “self” gDNA, but for qGT followed by algorithmic minor species determination of DF using the myTAI_HEART_ software (see Calculation of cfDNA Donor Fraction below). The qGT approach was also applied without modification to extracts of reconstructive genotypic mixtures used as reference materials in DF validation studies.

Before entering the qGT workflow, as for the bGT workflow, 15 ng extracted DNA (in this case post-transplant plasma cfDNA) was input into multiplexed high-fidelity library pre-amplification of regions including the myTAI_HEART_ informative and control target sites, performed on Eppendorf Mastercyclers^®^ followed by enzymatic cleanup. The qGT library amplification differed from the bGT library amplification in inclusion of low (0.2%), mid (1.0%), and high (10%) positive template controls (PTC’s) and a singular (HET) calibrator, but was otherwise equivalent. After Agilent 2100 Bioanalyzer amplification verification and pre-determined dilution, the amplified libraries, along with calibrators and controls, were subjected in duplicate to robotically controlled real-time Roche 480 PCR amplification/product detection using the allele-specific primers and probes also used for bGT.

The qGT step uses standard curves of heterozygous DNA sources to quantify alleles at each target. Quality metrics were developed to establish threshold acceptance criteria for a valid qGT run. These included comparisons to historic amplification shape, ≥ 75 targets with passing calibrators, log RNase P values ≥ 8 to ≤ 10, 70 passing quantifiable targets, ≥ 27 informative targets, a robust CV (rCV) ≤ 100%, skew (a metric designed to detect possible relatedness between donor and recipient) ≤ 1.01, and lot-specific acceptance criteria for PTC’s. Elevated rCV or skew would activate implementation of sample swap checks, and any high NTC’s would flag the reference lab manager to either institute the lab wipe testing protocol or possibly replenish with fresh reagents and repeat run.

### Calculation of cfDNA donor fraction

Validated output from the bGT and qGT runs was analyzed using the proprietary myTAI_HEART_ software. The original developmental version of this software (Method 1) integrated results of separate bGT reads for both recipient and donor at the 94 highly informative SNP targets of the myTAI_HEART_ test, using them to label the recipient and donor with three possible genotypes at each target (e.g. homozygous REF, heterozygous REF and VAR, and homozygous VAR). The donor and recipient bGT information was then inserted into the qGT analysis, along with standard curve results, to quantitate the allelic ratio, as a minor species proportion, at each target at the time of myTAI_HEART_ testing. The median of all informative and QC-passed allelic ratios was then used to determine DF.

The “Method 1” approach, with its utilization of independently determined recipient and donor genotypes, is not always practical given inconsistent availability of donor tissue/blood/DNA samples for genotyping. It served as an important gold standard for validation of an evolved method, Method 2, in which bioinformatics analysis of circulating post-transplantation cfDNA is able to distinguish “self” from “non-self” cfDNA without independent availability of donor genotyping. The evolved “no donor” Method 2 myTAI_HEART_ software algorithm, relying upon amplification results at the 94 highly informative bi-allelic SNP targets developed for Method 1, uses recipient bGT information in concert with the recipient’s qGT (post-transplant) cfDNA results to evaluate donor options in staged Monte Carlo simulations (greater than 30,000). Preliminary random selections of candidate donor genotypes simulate what DFs a given qGT sample could represent. Statistical analyses provide evidence of the most probable donor genotypes. Secondary Monte Carlo simulations further explore these likely donor genotypes and yield a range of probable qGT outcomes. Each simulation produces a candidate median DF value, along with quality control metrics. The final DF call provided on the myTAI_HEART_ test report is derived from the distribution of candidate DF values by a linear adjustment to the optimal midpoint. Validation Design and Results of the Method 2 “no donor” algorithmic approach are described in sections below.

### myTAI_HEART_ software validation design

Validation of the definitive “no-donor genotype” myTAI_HEART_ bioinformatic algorithm was predicated on validation of the original “with donor genotype” algorithm, both achieved using a cohort of 1168 heart transplant recipient samples. Within that cohort, 568 samples fell within the linear, quantifiable range of the original method (0.125–10% DF), and were randomly divided into two subsets (odd/even, when linearly arranged according to ascending DF), one subset to hone the new, “no donor” algorithm to the original “with donor” algorithm and the other subset to revalidate the results. This ensured a uniform distribution of high and low DF in each bin, while also distributing patient genotypes randomly. Comparison of DF results obtained using the previously validated “with donor” myTAI_HEART_ (Method1) algorithm versus the “no donor” (Method 2) algorithm was accomplished by Passing-Bablok regression analysis using R software to test hypothesis that the “no donor” method is equivalent to the predicate “with donor” method.

### Clinical validation design and study populations

#### Quantitative genotyping (DF) clinical validation design

A total of 452 whole blood samples obtained with prior informed consent from 88 volunteer adult and pediatric heart transplant recipient study participants between June of 2010 and Aug 2016 from two Milwaukee transplant centers—CHW and FMLH—were considered for inclusion in clinical validation of the myTAI_HEART_ assay of DF as an index of rejection probability. Organ procurement and transplantation arrangements were managed independently of the study for medical purposes through the United Network for Organ Sharing (UNOS) system in the United States under governance of the Uniform Anatomical Gift Act. Study approval was provided by the CHW Institutional Review Board (IRB), with approval for adult participants deferred to the CHW IRB by the Institutional Review Board of the Medical College of Wisconsin (MCW). Patients were recruited into this study from June 2010 through March 2013 with transplant procedures performed from June 2010 through July 2013. Patients were followed by biopsy and myTAI HEART testing through from June 2010 through Aug 2016. A preliminary account of this study population and initial results of application of the myTAI_HEART_ test to it were reported by members of our group as a Clinical Letter in the Journal of the American College Cardiology in 2018 [66]. Mean subject age at blood sampling was 12.7 ± 8.1 years (range 0.1 to 30.2 years); additional clinical demographics are here reported (see Results and discussion).

Clinical data was collected longitudinally on all study subjects, with focus on admission for transplant, treatment episodes for rejection, and all endomyocardial biopsy (EMB) procedures. All candidate whole blood samples for cfDNA analysis were collected in Streck BCT tubes and walked to the CRI Nucleic Acid Extraction Lab, where the two required serial 1400 x g x 10 min centrifugations were performed. Final supernatants immediately frozen and stored at -80°C were encoded by unique study identifiers prior to extraction per the TAI-approved TECAN automated protocol and transfer to the TAI Diagnostics Clinical Reference Lab for qGT according to the analytically validated myTAI_HEART_ protocol. Inclusion criteria for the clinical validation study included availability of clinical encounter and historical data, a properly collected and processed blood sample, a concurrent EMB specimen submitted for routine histopathologic evaluation and ISHLT 2004 grading by board-certified pathologists at the participating institutions, and access to the institutional EMB pathology report. The collection date and time of all blood samples paired with EMB were monitored to ensure blood samples were drawn immediately prior to any intra-cardiac access, not thereafter. Clinical exclusion criteria included blood sample collection less than 8 days after cardiac transplantation [88] or within 28 days of a rejection episode; concurrent mechanical circulatory support; a diagnosis of cancer or post-transplant lymphoproliferative disorder (PTLD) currently or within the last 2 years; pregnancy at the time of blood draw; or receipt of an allogeneic bone marrow or solid organ transplant (cardiac or non-cardiac) prior to the current cardiac transplantation. Pre-analytical sample exclusion criteria included delayed or improper whole blood centrifugation; extraction deviations from the approved myTAI_HEART_ protocol; and insufficient cfDNA yield for analysis. Analytical exclusion criteria included library failures and failure to pass analytical quality specifications. The clinical data content associated with all samples remained blinded to TAI Diagnostics personnel until all analytical data was generated and quality control metrics for inclusion/exclusion in the clinical validation data set were applied. A final set of 158 matched pairs of endomyocardial biopsy-plasma samples collected from 76 heart transplant recipients, both pediatric and adult, met study inclusion criteria and passed exclusion criteria.

Either the buffy coat from the cfDNA Streck tube collection or a separate EDTA tube for each study participant afforded gDNA for recipient bGT. A blood or tissue sample from the donor for each recipient participating in the study was available and similarly submitted for basic genotyping. This step allowed validation first of a “with donor genotype” myTAI_HEART_ protocol (Method 1) that would later be used to demonstrate equivalency of results produced by the “no donor genotype” myTAI_HEART_ protocol (Method 2) as described in the preceding section.

#### TCF concentration reference range in asymptomatic heart transplant patients, clinical validation design

For use in determination of the “normal” TCF reference range, Aim 1 of the DTRT prospective blinded multicenter study (NIH/NHLBI) provided a unique set of samples from asymptomatic heart transplant recipients. These samples were shipped to TAI Diagnostics and processed as described in *Methods*, *patient blood sample collection and plasma separation*. From a starting number of 2537 quality-controlled blood samples from 241 post-heart transplant recipient subjects [89,90], clinical exclusions were applied to identify samples from asymptomatic patients. Demographic features of the starting population were Sex: 148 (61.41%) male; Race: 8 (3.3%) Asian, 56 (23.24%) Black/African American, 158 (65.56%) Caucasian, 19 (7.88%) not reported; Ethnicity: 26 (10.79%) Hispanic or Latino, 196 (81.33%) non-Hispanic or Latino, and 19 (7.88%) not reported [89,90]. Samples from asymptomatic “healthy” subjects were conservatively chosen from this larger cohort by excluding samples classified as pre-cardiac transplant, associated with admissions for transplant surgery or rejection treatment, associated with a post-transplant re-admission, from patients who had died or had history of end organ dysfunction, from patients less than 2 months of age or less than 8 days post-transplant, from patients who had another transplanted, from patients who underwent post-transplant cardiac surgery or cardiac re-transplantation, and/or from patients with history of PTLD or cancer. Also excluded were samples collected +/- 7 days of angiography, +/- 28 days of readmission, +/- 30 days of treatment for infection or rejection or symptomatic diagnostic biopsy, +/- 30 days of fever, chest pain, shortness of breath, palpitations or other clinical symptoms; +/- clinical exam findings of S3, murmur, JVD, edema, respiratory or other findings; +/- 60 days of plasmapheresis, modified ultrafiltration, dialysis, mechanical circulatory support, or mechanical ventilation; +/- 60 days of diagnosis of ACR and/or AMR;+/- 6 months of cardiac arrest. After exclusions, 300 samples remained, 264 of which, from 106 subjects, had available TCF concentrations and thus constituted the selected pool of blood samples from asymptomatic “healthy” heart transplant patients. Normal reference range for TCF concentration in healthy heart transplant patients was determined from this group, using the average TCF concentration for each subject (see Results).

### Analytical validation design

All analytical validation protocols, covering multiple aspects of the myTAI_HEART_ testing protocol were performed according to recommendations of CLSI EP17-A2 Evaluation of Detection Capability for Clinical Laboratory Management Measurement Procedures [91] wherever applicable and in all cases consistent with expectations of CLIA and the College of American Pathologists (CAP).

#### TCF quantification—Analytical validation methods

Precision/reproducibility of extraction and quantification of TCF concentration was performed using five plasma samples prepared at cfDNA concentrations of 2ng/ml, 25ng/ml, 50ng/ml 100ng/ml and 200ng/ml. Extraction was performed in duplicate in each of 18 extraction runs using two lots of proprietary TAI extraction chemistry on two TECAN Freedom EVO 150 instruments by two technologists. Each extraction run contained a positive control, consisting of previously characterized plasma, and a negative extraction control; input sample plasma volumes were four ml. Resultant cfDNA was quantified by reference gene (RNase P) qPCR performed by two technologists using two reagent lots and two Roche LC 480 thermocycler systems. For the run to be valid, yield of the positive extraction control in ng cfDNA/mL plasma was required to fall within a previously established range. Runs that did not meet this requirement were removed from the analysis.

Limit of blank (LoB), limit of detection (LoD), and limit of quantification (LoQ) of TCF quantification were determined according to recommendations of CLSI EP17-A2 Evaluation of Detection Capability for Clinical Laboratory Management Measurement Procedures [91]. LoB was established using 4mL extractions of nuclease free water (Ambion, Cat# AM9932) as the sample source; nine replicates of nuclease free water were tested in each of six extraction and detection runs across four days. Two lots of reagents (extraction and RNase P) were used (three runs per lot). LoD was determined using four distinct plasma samples tested in replicates of five in each of six extraction and detection runs performed across four days using two reagent lot sets. LoQ was determined using contrived (spiked) plasma samples generated at concentrations of 5 ng/mL, 10 ng/mL, 15 ng/mL, and 20 ng/mL. In addition, unspiked plasma at ~2 ng/mL was also tested. Each concentration was tested in replicates of five in each of six extraction and detection runs performed across six days using two reagent lots (extraction and RNase P detection).

Linearity of TCF concentration quantification was assessed following recommendations provided in CLSI EP06-A Evaluation of the Linearity of Quantitative Measurement Procedures [92]. Contrived samples were generated at the following concentrations: 6,000 ng/mL, 4,000 ng/mL, 2,000 ng/mL, 1,000 ng/mL, 200 ng/mL, 150 ng/mL, 100 ng/mL, 75 ng/mL, 50 ng/mL, 25 ng/mL, 15 ng/mL, 10 ng/mL, 5 ng/mL, and unspiked (~2 ng/mL). Each sample was tested in duplicate in each of two runs. Runs were performed consecutively using the same lot of reagents and equipment. Samples that generated RNase P values above the highest standard curve point were diluted and retested such that they fell within the standard curve. The resulting quantifications were then multiplied by the appropriate dilution factor to back-calculate the starting concentration.

#### myTAI_HEART_ DNA fragmentation test—Analytical validation methods

Analytical validation of the myTAI_HEART_ DNA Fragmentation Assay was designed to individually establish performance characteristics of the short (≥115bp) and long (≥247bp) fragment *Alu* amplification tests that together comprise the myTAI_HEART_ DNA Fragmentation Assay. LoB, LoD, LoQ, precision, accuracy, and linear range of the ALU115 and ALU247 amplifications were determined such that those characteristics in the resultant *Alu* ratio could be implied.

To support myTAI_HEART_ DNA Fragmentation Assay validation studies, as described above in *Reference Materials*, Covaris-sheared human buffy coat gDNA spiked back into aliquots of paired plasma was used to produce plasma samples at targeted long to short DNA fragmentation ratios of 0.2 to 0.5, yielding final actual ratios of 0.19 to 0.490. Additionally, for studies not linked to extraction, gDNA was introduced into 0.1X Tris-EDTA buffer. Samples with extraction were quantified by RNase P qPCR on a Roche LC480 prior to use in determinations of precision, DNA fragmentation assay LoB, LoD, LoQ, and linearity according to CLSI guidelines [91,92], see Results and discussion. Specified acceptable ranges for individual standard curve amplification efficiencies and analytical measurement ranges were defined.

#### Basic genotyping (bGT) and quantitative genotyping (qGT)—Analytical validation methods

Reproducibility of the bGT protocol was assessed using a panel of 6 patient whole blood samples from 6 different individuals that were each aliquoted and extracted in five DNA extraction runs followed by library amplification and running of each library through six bGT runs targeting 94 SNP targets + 2 controls performed by two technologists over a span of 6 days.

Analytical performance characteristics of the essential qGT portion of the myTAI_HEART_ test that generates plasma cfDNA DF were determined using a series of studies designed to determine Limit of Blank (LoB), Limit of Detection (LoD), Limit of Quantification (LoQ), Linearity, and Precision. Also evaluated were potential interfering effects of substances commonly present in the circulation of heart transplant patients, as well as carryover and cross contamination.

For LoB, sheared gDNA isolated from blood of 16 different individuals was used to simulate 16 transplant patients with no donor DNA present. Overall assay LoB was determined by testing between one to four libraries for the 16 samples across two runs. Each sample library was analyzed through the myTAI_HEART_ algorithm using eighteen different donor pairings resulting in 18 data points per library tested. Samples that did not meet QC criteria were removed from analysis.

For LoD, nine distinct reconstruction samples were made at a theoretical 0.1% donor fraction, a low-level fraction just above the LoB. Each sample, at an input of 15ng cfDNA was amplified in triplicate in each of three library runs and then subjected to qGT. Each library run of 27 samples (nine reconstruction samples run in triplicate) was tested on each of three days with a distinct reagent lot, for a total of three days and three reagent lots. Samples that did not meet QC criteria were removed from the analysis. LoD was determined for each reagent lot by using the equation LoD = LoB + Cp*SDl as outlined in CLSI EP17-A2 [91].

For LoQ, nine additional distinct reconstruction samples were made at theoretical DFs of 0.2% and 0.3% using the same sample reconstruction pairings used in the LoD study above. Samples of each were amplified in triplicate in three separate library runs over three days, with a distinct reagent lot each day, followed by quantification. Samples that did not meet QC criteria were removed from the analysis. Data generated from the LoQ runs were combined with the data from the LoD runs (at 0.1% DF). For each reconstructed sample, data from all three library runs was pooled to generate the %CV of DF. The average %CV for each DF fraction was plotted to generate a precision profile curve to estimate the concentration at which the precision of the assay is ≤ 20%CV.

For testing of linearity of DF determination, three distinct series of contrived samples were constructed at 0.10, 0.20, 0.30, 0.40, 0.50, 0.75, 1.00, 1.50, 2.00, 4.00, 6.00, 8.00, and 10.00% DF using gDNA as described in Materials and Methods. Within each series, samples were tested through the myTAI_HEART_ testing workflow in duplicate and processed in the same run with a single lot of reagents. DF results were plotted against the theoretical DF intended by the reconstructions and assessed for linearity according to CLSI EP06-A Evaluation of the Linearity of Quantitative Measurement Procedures. Samples that did not meet QC criteria were removed from analysis.

For evaluation of precision of DF determination, three reconstruction samples were generated at low (~0.20%), medium (~1.0%), and high (~10%) DF. Samples were mixed and aliquoted into single use aliquots and tested in a single replicate up to two times a day over 31 days for a total of 46 runs (46 replicates of each samples). Multiple operators tested samples across several reagent lots and equipment lines. Samples that did not meet QC criteria were removed from the analysis. The %CV within run date and across all samples was determined for each sample.

Accuracy of myTAI_HEART_ bGT and qGT results was verified by comparison to results obtained by Illumina sequencing as an independent second method. For bGT, eight amplified DNA libraries across four control samples were used for comparison, and for qGT six amplified DNA libraries across three control samples (see Results). Sequence processing was performed using CentOS 7.4, bwa 0.7, Trimmomatic-0.38, samtools 0.1, bcftools/htslib 1.9, GRCh37/hs37d5, bam-readcount 0.8, Snakemake 5.3, Python 3.6, RStudio 1.0, R 3.4, R-xlsx 0.6, and plyr 1.8. Variant call quality was filtered at Phred+30 in bcftools.

#### Interfering substances—Analytical validation methods

Parallel sets of studies, identical in fundamental design, were executed to assess the effects of potentially interfering substances on four individual aspects of the myTAI_HEART_ test: the myTAI_HEART_ DNA Fragmentation Assay, quantification of TCF concentration, basic genotyping, and qGT (DF determination). Ten substances were chosen for the study, including bilirubin, hemoglobin, EDTA, prednisone, tacrolimus, sirolimus, mycophenolate, cyclosporine A, triglycerides, and IVIg, these representing endogenous substances commonly elevated in plasma samples from heart transplant recipients as well as exogenous substances commonly introduced by standard medical therapies. Additionally, for extraction and genotyping validations, potential interference by two viruses, cytomegalovirus (CMV) and BK virus (BKV) was tested. Concentrations for each substance/virus were selected according to CLSI EP07-A2 [93], or previously published literature where appropriate, and are given in S1 Table. Aliquots of individual patient and contrived patient samples prepared according to needs of each tested aspect of the assay (see sample preparation details below) were spiked with the potentially interfering substances, and, where indicated, the CMV and BK viruses, each in isolation. Aqueous or organic solvents required to dissolve a substance during their preparation (*e*.*g*., nuclease free water, ethanol, DMSO) were tested separately in the absence of that substance. Samples were then extracted in triplicate using the myTAI_HEART_ automated extraction procedure prior to processing through the intended myTAI_HEART_ workflow. Any samples not passing QC criteria during testing were removed from analysis. Passing results were analyzed using the statistical software package JMP, version 14 (SAS Institute, Inc., Cary, NC) using the Tukey-Kramer HSD test following one-way ANOVA testing to determine if the results from exposed samples deviated significantly from those of paired samples extracted and tested without spiked-in substance.

Sample preparation protocols and logistics unique to each interfering substance application are as follows: Interference studies for TCF quantification and myTAI_HEART_ DNA Fragmentation Assay analyses were performed in concert using three separate contrived human plasma samples prepared at TCF concentrations targeted at 2ng/ml, 25ng/ml, and 50ng/ml, each possessing a slightly different *Alu* ratio. bGT interference testing was performed using blood samples from two individuals. Interference testing of the qGT DF determination step employed two separate contrived plasma samples prepared by mixing plasma from two previously genotyped individuals in different proportions to simulate plasma from two transplant patients with DFs near the cut point for increased risk of rejection. All prepared samples were immediately frozen in single use aliquots, then thawed and immediately spiked, extracted in triplicate and processed through the intended workflows and statistical analyses of results as outlined above.

#### Carryover/cross-contamination—Analytical validation methods

Potential carryover/cross-contamination during extraction and downstream analytical workflows that could impact results of TCF quantification and myTAI_HEART_ DNA Fragmentation analyses was assessed by testing high positive contrived samples (see Reference Materials) generated at ~200 ng cfDNA/mL alongside negative (nuclease free water) samples in a 32 position checkboard pattern on the TECAN instrument across two independent runs. The extracted samples maintained the same sample positioning during subsequent RNase P quantification of TCF and myTAI_HEART_ DNA Fragmentation testing (ALU115 and ALU247).

Potential carryover/cross-contamination during extraction and all downstream analytical workflows that could influence DF results was assessed using contrived plasma generated by mixing plasma from two unique individuals together in two different proportions to simulate plasma samples from two transplant patients, one with low (~0.2%) DF and one with high (~2.0%) DF, aliquoted into single use, 2mL portions and frozen at -80°C. Samples were subsequently thawed and extracted in a checkboard pattern in two independent TECAN runs. The extracted samples maintained the same sample positioning during subsequent processing in duplicate through the full myTAI_HEART_ qGT workflow to produce DF results.

## Results and discussion

### A clinically validated laboratory-developed multiplexed, high-fidelity amplification qGT test (myTAI_HEART_) for monitoring of heart transplant rejection without donor genotype

myTAI_HEART_ is a laboratory developed test (LDT) developed for clinical diagnostic performance exclusively in the College of American Pathologists (CAP) and Clinical Laboratory Improvement Amendment (CLIA)—accredited TAI Diagnostics Clinical Reference Laboratory. The entire assay and workflow presented here, from whole blood sample collection and plasma separation through bioinformatics analysis and result reporting, depicted at high level in Fig 1, was locked and captured in formal standard operating and quality management procedures prior to analytical validation according to approved Validation Plans, findings summarized herein.

The test uses a panel of 94 highly informative SNPs to quantitatively genotype cfDNA in the patient’s plasma, accurately distinguishing “donor specific” cfDNA originating from the engrafted heart from “self-specific” cfDNA originating from the recipient’s native cells and reporting the cfDNA DF as a direct marker of selective injury to the transplanted organ. It is intended to aid in categorization of the patient as at low or increased risk of moderate (grade 2R) to severe (grade 3R) ACR at the time of testing in conjunction with standard clinical assessment. This test is indicated for use in heart transplant recipients who are 2 months of age or older and ≥ 8 days post-transplant based upon study populations of wide age range that extend into the very early transplant period demonstrating lack of age and sex bias and typical rapid return of DF level to baseline within 4–7 days post-transplant [88]. It is currently restricted to use in single organ post-heart transplant patients and is contraindicated in patients who:

- are pregnant
- currently have or in the past have had another transplanted organ (solid organ or allogeneic bone marrow)
- have post-transplant lymphoproliferative disease
- have cancer or have had cancer within the previous 2 years
- are on mechanical circulatory support
- are closely related to the transplant donor

As with negative EMB results, a heart transplant recipient with a negative myTAI_HEART_ result should continue to be monitored according to standard clinical care, with all results interpreted in the context of the patient’s overall clinical findings, history, and laboratory results. A conservative cut-off value with 100% NPV for increased probability for moderate to severe ACR, established using 158 matched pairs of endomyocardial biopsy-plasma samples collected from 76 heart pediatric and adult transplant recipients, as described in Methods, Clinical Validation Plan and Study Populations, was purposefully selected. Clinical and analytical test performance characteristics (see Results, Clinical Performance Characteristics) strongly support the test’s intended use as a noninvasive, sensitive means of ruling out significant cardiac transplant rejection with confidence, particularly important for patients for whom biopsy is not a good current option and fulfilling a critical, previously unmet need in infants and children.

In the clinical validation study dataset of 158 matched blood-EMB pairs, 95% of the 94 SNP targets used for myTAI_HEART_ determination of DF were individually found to be informative in more than 30% of patients, with mean informativity rate of 36.6%. This compares favorably to a theoretical maximum of 37.5% at maximal diversity (SNP minor allele frequency = 50%). The myTAI_HEART_ SNP target panel is statistically indistinguishable from optimal. In reference to Table 1, we conclude that no target in the myTAI_HEART_ panel is less than 11% frequent in any major population group identified by the largest genomic dataset to date. In more finely divided groups (Table 2), some targets may be less than 10% frequent, but in every population studied at least 90% of our panel is >25% frequent.

**Table 1:** Summary statistics across 94 targets for minor allele frequency (MAF) as presented in the GnomAD [77] 2019 database.

Population Group	Subj >= N	Proportion > 25% MAF
African/African American^1^	4,359	0.947
Ashkenazi Jewish	145	0.936
East Asian	780	0.904
European Finnish	1,738	0.946
European Non-Finnish	7,718	0.947
Latino	424	0.936
Other	544	0.979
Altogether	15,708	1.000

^1^This genetic supergroup represents the greatest diversity among modern humans and includes representatives across their geographic range including, the subgroups, Nigerian Yoruba, Kenyan Luhya, Western Gambians, Sierra Leone Mende, Nigerian Esan, SW US African Americans, and Barbados Africans.

**Table 2:** Subgroups from the 1000 genomes project [78] providing further distinction in the African/African American population.

Population Group	Subject N	Proportion > 25 MAF
Yoruban	88	0.915
Luhya	97	0.926
American	61	0.957

TCF concentration is necessarily determined as part of the measurement of DF and independently conveys important information often useful in clinical evaluation of heart transplant patients. Quantitative reporting of this concentration was thus validated as part of the overall myTAI_HEART_ validation plan, enabling its quantitative reporting along with DF within the myTAI_HEART_ test report. A reference range for TCF concentration in asymptomatic heart transplant patients of 0.91–37.70 ng/mL plasma (95% reference interval) was established based upon a study of 241 heart transplant patients, 106 of which were determined to be “healthy” and asymptomatic at the time of testing (see Methods, Clinical Validation Plan and Study Populations).

**Fig 4:**
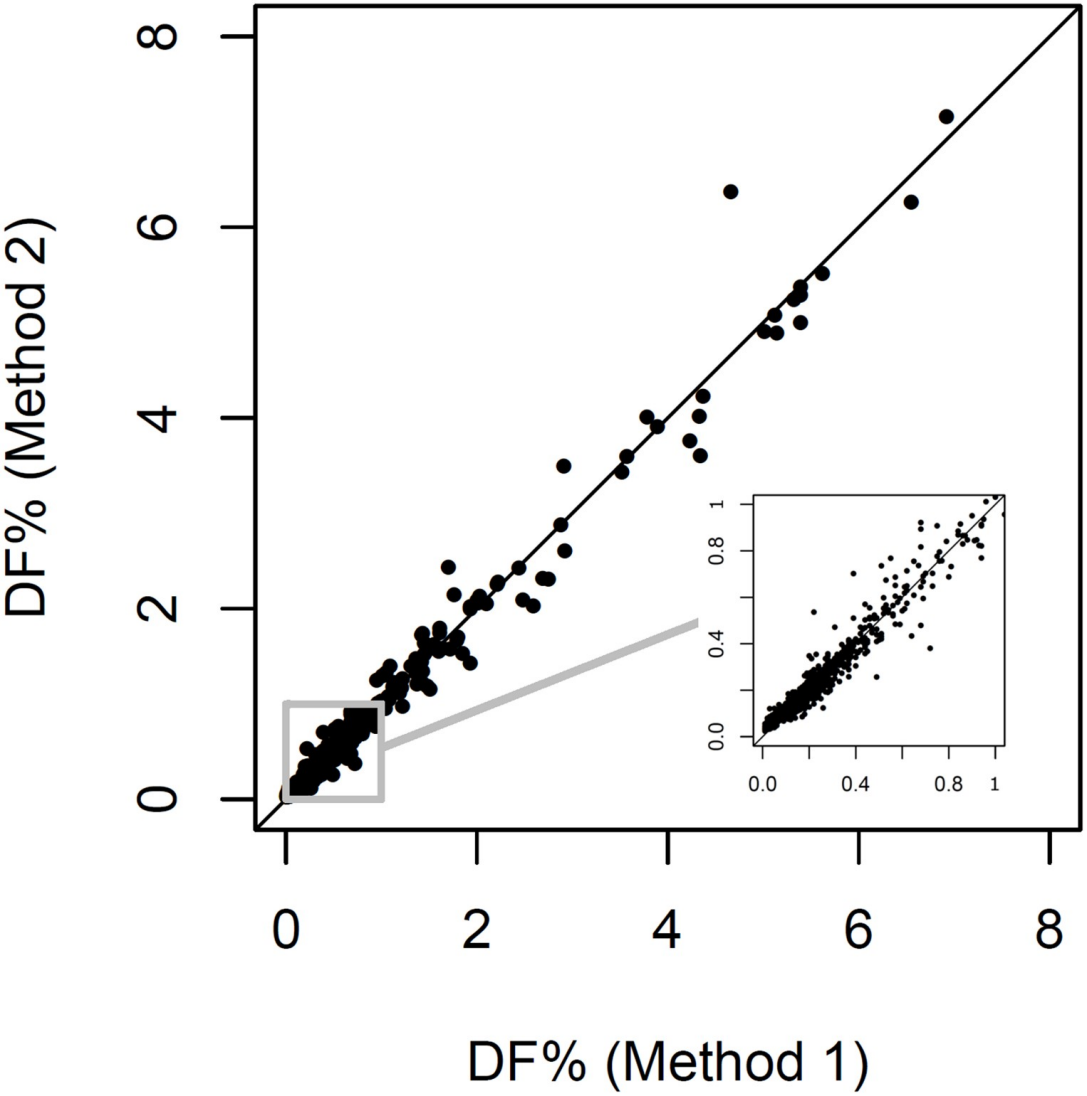
Equivalence of the “no donor genotype” algorithm for DF determination. Samples passing QC used in creation of the “no donor genotype” algorithm are shown (N = 1128). Inset magnifies the 0–1% range. Line shows equality.

As described in Methods & Materials (myTAI_Heart_ software validation design), tuning of the myTAI_HEART_ software for use without requirement for donor genotyping was performed on a training data set to hone the “without donor genotype” (Method 2) algorithm to the previously validated “with donor genotype” (Method 1) algorithm. The Method 1 and honed Method 2 algorithms were then individually applied to a separate validation data set. Results for 1128 of 1168 samples passed QC for both methods and are shown in Fig 4. To assess statistical equivalency of the Method 1 and 2 results, samples within the Fig 4 subset falling within the DF linear range of the test (N = 444 with 0.165% < DF < 10%) were subjected to Passing-Bablock linear regression analysis. Acceptance of equivalency required that the confidence interval of the slope contain 1 and that the confidence interval of the intercept contain 0. The prescribed acceptance criteria were met (Table 3), demonstrating that DF results produced by the “no donor” method are statistically equivalent to those of the predicate “with donor” method (Fig 4).

**Table 3:** Passing-Bablock linear regression results, Method 1 (with donor genotype) algorithm vs Method 2 (without donor genotype) algorithm.

Parameter	Point Estimate	Lower Confidence Interval	Upper Confidence Interval
**Intercept**	0.004	-0.003	0.010
**Slope**	0.993	0.979	1.010

### Sample handling—A critical determinate of DF validity

Low percentages of donor-specific cfDNA (<0.3%) within an already minute total population of cfDNA molecules are observed in most heart transplant recipients (typically less than 10 ng/ml) and necessitate rigorous prevention of leukocyte gDNA contamination when preparing plasma for cfDNA DF analysis to prevent underestimation of DF. Potential causes of contamination include leukocyte lysis or exocytosis during prolonged or otherwise stressful exposures during sample collection and processing; incomplete removal of leukocytes from the plasma phase of whole blood prior to plasma freezing and shipping for off-site cfDNA analysis; and, more rarely, immediate pre-phlebotomy events, such as drug infusions, that may in some instances cause transient leukocyte lysis. Failure to exert the needed pre-analytical precautions in cfDNA DF testing will result in underestimation of DF and, potentially, false negative reporting of rejection risk. A consistent recommendation for effective leukocyte elimination from plasma is inclusion of two sequential centrifugation steps. [94–97]

Minimization of leukocyte gDNA contamination is also important in cfDNA analyses applicable in fetomaternal medicine and oncology, although the impact of low-level leukocyte contamination is generally better tolerated in those non-transplant applications due to the higher TCF levels and higher minor species excluding early or minimally residual tumors very low fetal cfDNA representations. For instance, TCF levels average as much as 20x higher in unselected cancer patients [98] than in normal subjects and most heart transplant patients, and during pregnancy the percentage of cfDNA in maternal plasma is roughly 10% median in the first two trimesters [99].

For all cfDNA applications, the plasma cfDNA component of diagnostic interest can be protected from both degradation and leukocyte gDNA contamination by select blood collection additives. EDTA is preferred over citrate and heparin as an anticoagulant, in part, because EDTA salts, in addition to protecting cfDNA by preventing release of cellular DNA through coagulative stress, inhibit *ex vivo* DNase activity [100]. Many studies have demonstrated stability of cfDNA within unspun whole blood samples without significant contamination by leukocyte gDNA when collected in K2-EDTA tubes and held at RT for 4–6 hrs prior to plasma separation by centrifugation [101,102,103,104,105,106,107]. That time can be extended up to 24 hrs by refrigeration at 4°C [100,108]. After several hours at RT, TCF in the plasma fraction of EDTA blood tubes without addition of stabilizers begins to increase rapidly due to leukocyte lysis and/or release of extracellular vesicles from intact leukocytes [109], thus requiring plasma separation by centrifugation within 6 hrs after phlebotomy (4 hrs for added margin of safety) unless refrigerated.

To extend the window of stability of EDTA anticoagulated whole blood samples for cfDNA analysis and add the convenience of shipping to reference laboratories prior to shipping, a plethora of commercially available cell preservation tubes containing proprietary cell membrane stabilizing additives that inhibit release of cellular DNA for prolonged periods over a wide temperature range have been developed. Pre-dating these developments, simply adding formaldehyde, a widely used cross-linking fixative, not surprisingly was found to be effective [110,111]. Formaldehyde was rejected as a viable option upon realization that it introduces non-reproducible sequence alterations in DNA and heavy modifications in the poly(A) tail of mRNA, both deleterious to downstream analyses; and also makes extraction more difficult [112]. Subsequently, several commercial offerings of dedicated cell-stabilizing blood collection tubes claiming to be “formaldehyde-free” or “fixative free” have been introduced, beginning with Cell-Free DNA blood collection tubes (BCT^®^) (Streck, Omaha, NE, USA) in 2010 [109]. These have been followed by tubes with various proprietary preservative formulations, including Cell-Free DNA Collection Tubes (Roche Diagnostics, Pleasanton, CA), PAXgene^®^ Blood ccfDNA Tube (PreAnalytiX GmbH, Hombrechtikon, Switzerland), LBgard™ Blood Tube (Biomatrica, Inc., San Diego, CA, USA), cf-DNA Preservative Tube (Norgen Biotek, Thorold, ON, CA), Blood Stasis™ 21-ccfDNA Tube (MagBio Genomics, Gaithersburg, MD, USA), and Blood Exo DNA ProTeck^®^ tubes (CFGenome LLC, Denver, CO, USA) among others, recently reviewed [97]. Some types of preservative tubes, particularly the early entry Streck BCT tubes, have been widely utilized as a means of whole blood sample transport at ambient temperatures to reference laboratories for delayed plasma separation and cfDNA analysis. Each stabilizing option requires investigation to support appropriateness for the demands of any specific clinical diagnostic or investigative application. Potential unintended negative consequences of use of such stabilizers, in addition to effectiveness in cellular membrane stabilization under real-life shipping conditions, must be characterized and understood.

We previously studied the effect of 72 hr temperature-monitored shipping of whole blood by air courier in Streck BCT tubes for prenatal testing purposes, finding them generally acceptable, but noting an increase in TCF concentration and a decrease in fetal fraction in samples shipped on cool packs that lowered temperature within insulated containers into the 4–7°C range [113]. Unexpected effects of storage temperatures on cfDNA collected in Streck BCT tubes were also reported by Medina Diaz et al [114] who observed up to 10-fold increase in longer gDNA fragments during 3–5 day storage at either 4°C or 40°C in Streck BCT tubes, accompanied by decreased average plasma volume, compared to the same storage duration in BCT tubes at RT or to storage in standard K2-EDTA tubes for 2 hr at RT. This constituted a 2-fold dilution of circulating tumor-specific DNA in that study and greater than 60% presence of long wild-type DNA (402:96 bp ratio of 0.6) [114]. Notably, this effect was observed only a few degrees outside the manufacturer’s stated stability range of 6°C to 37° for up to 14 days of storage [115]. High quality plasma for cfDNA analysis should have a low proportion of long DNA fragments, ranging from 0.2 to 0.4 in our own studies, here reported, to those of others [101,114]. Although acceptable long-fragment percentages will vary somewhat with lengths of DNA fragmentation target pairs employed, a 0.6 402:96 bp ratio would be problematic for cfDNA DF analysis.

**Fig 5:**
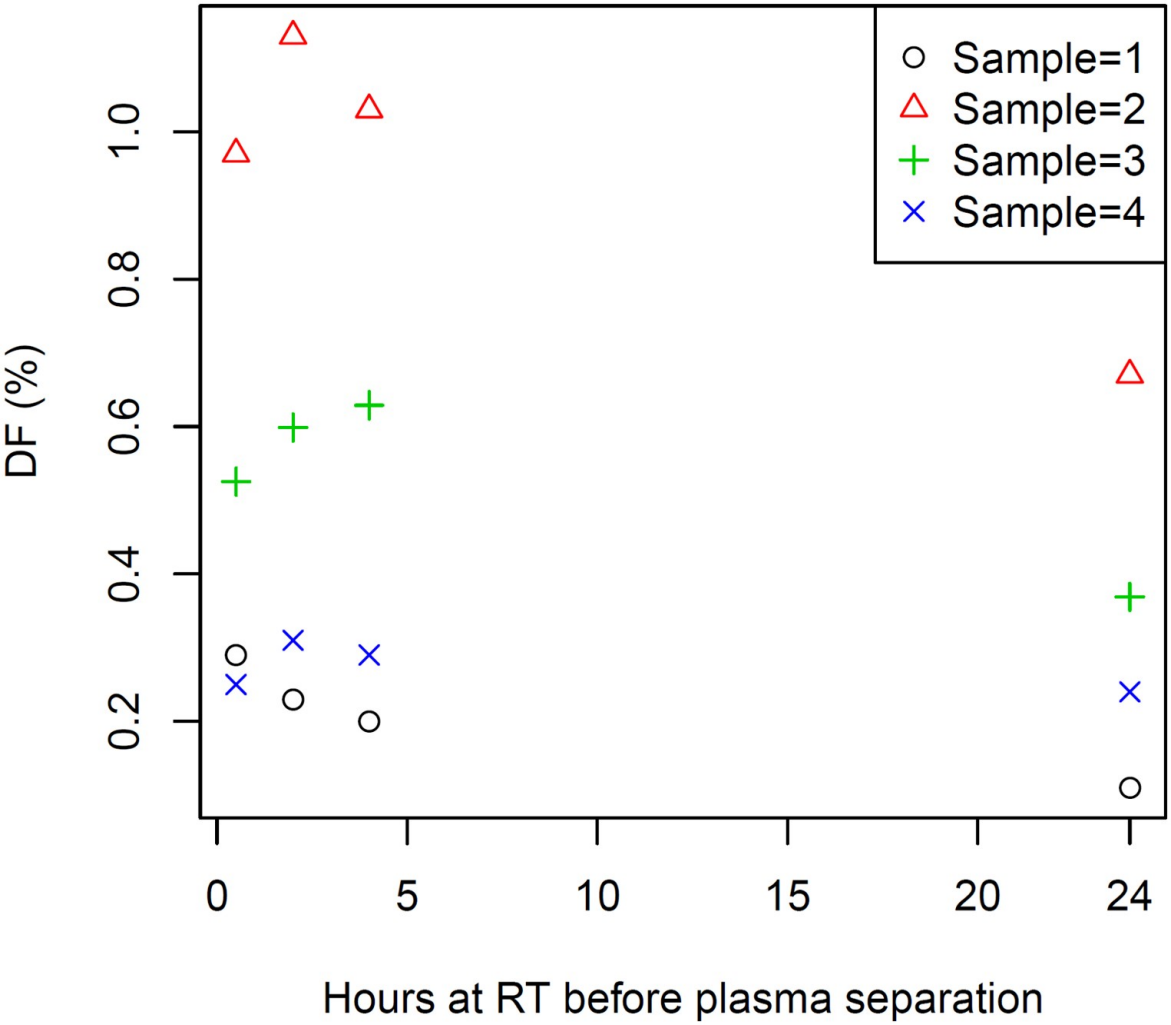
Effect of RT whole blood storage duration (0–24 hr) in Streck BCT tubes on myTAI_HEART_ cfDNA DF. Significant drop in plasma cfDNA DF (0.2% +/-0.05% per day, p<0.01) was observed in four manufactured BCT whole samples (each at a unique starting DF) when plasma separation was delayed by 24 hrs at RT post-phlebotomy. DF is highly sensitive to cfDNA dilution by even very low levels of leukocyte lysis prior to plasma separation. See text for methodological detail.

In light of unpredictable shipping variables (climates and altitudes), the temperature sensitivities of BCT tubes, while potentially tolerable in some cfDNA applications not requiring sensitive assessment of minor species proportions, can have significant impact on measurement of low cfDNA DF’s generally observed within already low TCF complements in heart transplant patients. Adding to this concern, we became aware during recent clinical trials employing BCT tubes for air transport shipment of whole blood samples from heart transplant patients, that even with protection afforded by Styrofoam-insulated and gel-pack-protected shipping containers, inconsistent myTAI_HEART_ DF results were observed with shipped whole blood samples. In contrast, more consistent results were obtained using shipped samples for which plasma was separated from BCT tubes and frozen quickly at the collection site before shipping on dry ice according to TAI protocol [89]. To investigate further, we studied the impact on DF determination of 0.5 hr, 2 hr, 4 hr, and 24 hr RT incubations prior to plasma isolation from BCT tubes and entry into the myTAI_HEART_ protocol. This was accomplished using manufactured “post-transplant” whole blood samples from four healthy donors drawn into Streck BCT tubes and promptly spiked within 20 min of phlebotomy with approximately 1 ng of “donor” cfDNA previously isolated from a commercial blood lot (see Materials and methods, Reference Materials). We observed statistically significant reduction in DF when plasma preparation was delayed by 24 hrs at RT post-phlebotomy (Fig 5).

The myTAI_HEART_ test is designed to provide high-level sensitivity required for detection of acute cellular rejection in heart transplant patients, a task more demanding than detection of acute antibody-mediated rejection in these patients [89]. This requires high quality plasma to minimize potential dilution of donor-specific cfDNA with leukocyte gDNA and is readily achievable by rapid processing of whole blood to purified plasma by two low speed spins in standard unrefrigerated swinging bucket centrifuges within 2–4 hrs of collection per TAI protocol. The purified plasma is then frozen and shipped overnight on dry ice to TAI Diagnostics for cfDNA analysis. For clinical diagnostic use, plasma separation at the collection site is facilitated by use of provided plasma separator K2-EDTA plasma preparation tubes (PPT) for the first, most critical spin, allowing consistent, convenient pour off of plasma by general medical technicians and technologists.

### Validation of a clinical DNA fragmentation assay for quantitative monitoring of pre-analytical contamination of cfDNA with leukocyte gDNA

To validate *Alu* ratio for quantitative quality control use in detection of significant leukocyte lysis in clinical samples submitted for cfDNA analysis, it was necessary to construct combinations of un-sheared and sheared gDNA to produce clinically relevant target *Alu* ratios in a range of ALU115 and ALU247 concentrations (1.56 pg/μl to 100 pg/μl, see Reference Materials). For some validation studies (*e*.*g*., ALU115 and ALU247 linearity, precision, LoQ) for which extraction was not required, human gDNA from a commercial vendor was used directly to make defined gDNA concentrations ranging from 0.25 pg/μl to 400 pg/μl in 0.1X TE Buffer. For other validation studies requiring DNA extraction, contrived samples prepared by spiking combinations of sheared and unsheared gDNA into aliquots of human plasma were employed (see Methods, Reference Materials).

Automated extraction of cfDNA from 4 ml volumes of contrived plasma samples prepared for this validation was performed on TECAN Freedom EVO 150 liquid handlers using proprietary chemistry, followed by quantification by RNase P qPCR according to clinically validated protocols herein described.

#### Precision/LoB/LoD/LoQ, myTAI_HEART_ DNA fragmentation assay

Precision of ALU115 and ALU247 qPCR measurements was determined using commercially available gDNA diluted in 0.1X Tris-EDTA buffer to target concentrations of 100, 50, 25, 12.5, 6.25, 3.13, 1.56 pg/μl. Each dilution was tested for ALU115 and ALU247 amplification in duplicate wells per run, two runs per day for ten days, totaling 40 measurements for each dilution (Tables 4 and 5).

**Table 4:** Precision results, ALU115 qPCR.

Target cfDNA Concentration (pg/μl)	Total n	Measured Average cfDNA Concentration (pg/μl), ALU115 qPCR	Standard Deviation	%CV Estimate	Lower %CV	Upper %CV
**100**	40	99.1	14.7	14.8	12.1	19.1
**50**	40	46.9	6.00	12.8	10.4	16.5
**25**	40	22.9	3.54	15.5	12.6	20.0
**12.5**	40	12.8	1.50	11.7	9.6	15.1
**6.25**	40	6.57	1.22	18.7	15.2	24.2
**3.13**	40	3.18	0.45	14.1	11.5	18.2
**1.56**	40	1.68	0.33	19.9	16.2	25.8

**Table 5:** Precision results, ALU247 qPCR.

Target cfDNA Concentration (pg/μl)	Total n	Measured Average cfDNA Concentration (pg/μl), ALU247 qPCR	Standard Deviation	%CV Estimate	Lower %CV	Upper %CV
**100**	40	96.5	10.97	11.4	9.3	14.6
**50**	40	47.9	8.51	17.8	14.5	23.0
**25**	40	23.1	3.00	13.0	10.6	16.8
**12.5**	40	12.7	1.74	13.7	11.2	17.7
**6.25**	40	6.37	0.96	15.0	12.2	19.4
**3.13**	40	3.19	0.62	19.3	15.7	25.1
**1.56**	40	1.69	0.42	24.8	20.1	32.4

LoB values for the myTAI_HEART_ DNA Fragmentation Assay ALU115 and ALU247 fragment analyses were individually determined using 0.1X TE as the sample source. Twelve replicates were tested in eight runs and performed twice per day across four days. Two lots of 0.1X TE were used for a total of 95 measurements (each) for the ALU115 and ALU247 amplifications. Resultant distributions of blanks for both ALU115 (S2 Fig) and ALU247 (S3 Fig) did not display a normal fit. The nonparametric option for obtaining LoB was used per CLSI EP17-A2 Evaluation of Detection Capability for Clinical Laboratory Management Measurement Procedures [91], assigning the final LoB for ALU115 as 0.014 pg/μl and the final LoB for ALU247 as 0.006 pg/μl, each representing the greater of the LoB values determined for the two tested 0.1X TE lots (Table 6). For determination of LoD values of the short and long fragment components of the myTAI_HEART_ DNA Fragmentation Assay, human gDNA (see *Reference Materials*) was diluted in 0.1X TE to concentrations of 4, 2, 1, 0.5 and 0.25 pg/μl. Each resultant sample was tested in five wells per run and two runs per day for four days yielding a total of 40 separate measurements collected across eight runs for each fragment length. Two lots of primers and probe were tested. LoD values for each assay (ALU115 and ALU247) were determined using the parametric approach as outlined in CLSI EP17-A2, pages 16–17 [91]. As the %CV for all of these low-level tested samples was < 30%, statistics for the 0.25 pg/μl sample were used to perform LoD calculations. The resultant LoD is 0.122 pg/μl for ALU115 and 0.126 pg/μl for ALU247, representing the greater values determined for the two reagent lots (Table 6).

**Table 6:** Limit of detection results, ALU115 and ALU247.

	*Alu 115 Assay*		*Alu 247 Assay*	
	Reagent Lot A	Reagent Lot B	Reagent Lot A	Reagent Lot B
**SD**_**L**_	0.0654	0.0655	0.0728	0.053
**n**_**i**_	40	40	40	40
**J**	1	1	1	1
**c**_**p**_	1.656	1.656	1.656	1.656
**L**	40	40	40	40
**LoB**	0.014	0.009	0.005	0.006
**LoD**	0.122	0.117	0.126	0.093

For determination of LoQ for the short and long fragment components of the myTAI_HEART_ DNA Fragmentation Assay, human gDNA prepared as described in Materials and Methods (*Reference Materials*) was diluted in 0.1X TE to concentrations of 4, 2, 1, 0.5 and 0.25 pg/μl. Each sample dilution was tested in five wells per run and two runs per day for four days, producing 40 measurements collected across eight runs for each fragment length. Two lots of primers and probe were tested (Reagent Lot A and Reagent Lot B). LoQ for each assay was determined according guidelines outlined in CLSI EP17-A2 [91]. The LoQ for the short and long fragment assays were determined as follows using the data shown in Table 7: The mean and SD for the lowest level sample tested were calculated across all replicates for each reagent lot. The Bias was calculated by subtracting the assigned value (0.25 pg/μl) from the mean. The “TE” value was then determined using the equation TE = Bias + 2*SD. Since the TE values calculated from both the short and long fragment data sets for the 0.25 pg/μl sample were < 30%, the LoQ for both assays was determined to be 0.25 pg/μl.

**Table 7:** Limit of quantitation results, myTAI_HEART_ DNA fragmentation assay.

*Concentration (pg/μl)*		*ALU115 Assay*			*ALU247 Assay*		
		Reagent Lot A	Reagent Lot B	Pooled	Reagent Lot A	Reagent Lot B	Pooled
**4**	**Average**	4.010	3.732	3.957	3.616	3.732	3.674
	**SD**	0.912	0.692	0.801	0.778	0.569	0.675
	**%CV**	23	18	20	22	15	18
**2**	**Average**	2.013	2.037	2.025	1.857	2.002	1.929
	**SD**	0.401	0.354	0.373	0.254	0.329	0.299
	**%CV**	20	17	18	14	16	15.5
**1**	**Average**	1.069	1.051	1.060	0.971	1.022	0.996
	**SD**	0.254	0.222	0.236	0.180	0.212	0.196
	**%CV**	24	21	22	19	21	19.6
**0.5**	**Average**	0.538	0.532	0.535	0.479	0.4992	0.489
	**SD**	0.121	0.114	0.116	0.079	0.097	0.088
	**%CV**	23	21	21.7	17	19	18
**0.25**	**Average**	0.286	0.283	0.285	0.255	0.264	0.259
	**SD**	0.065	0.066	0.065	0.073	0.053	0.063
	**%CV**	23	23	23	28.6	20	24.2

**Fig 6:**
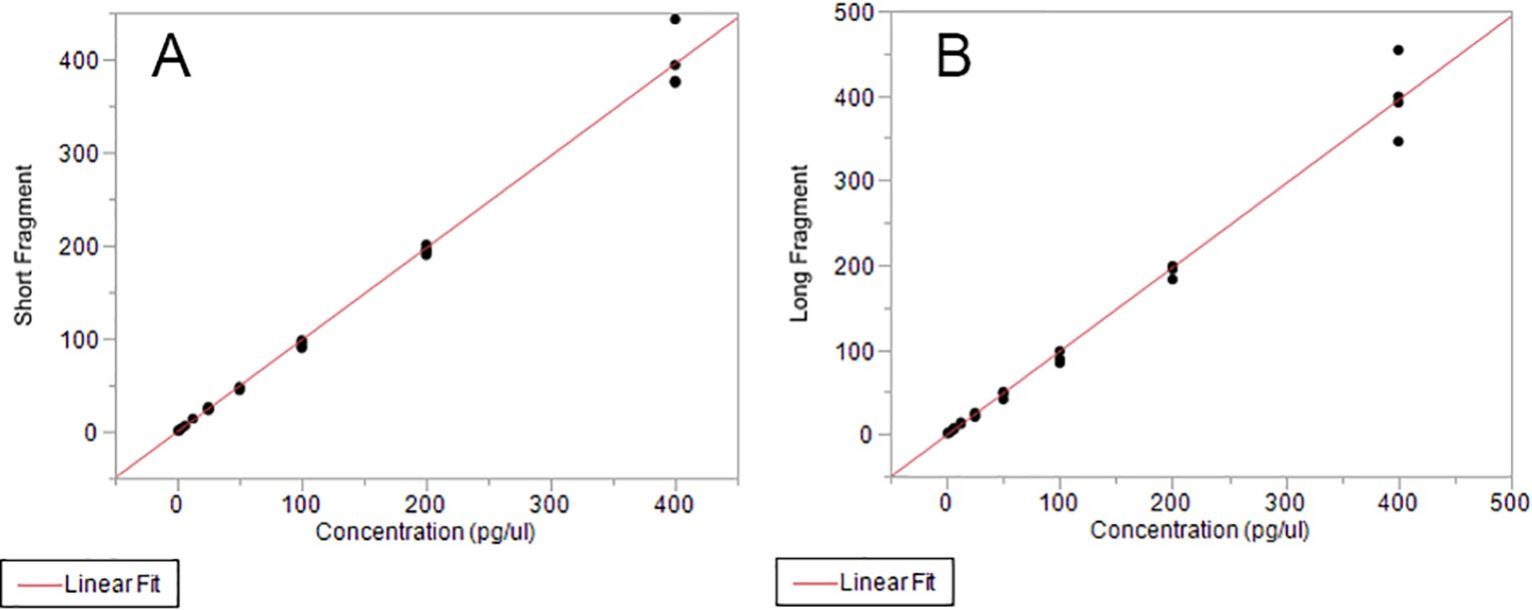
DNA fragmentation assay linearity results. (A) ALU115 (B) ALU247, see Table 8 for statistical data.

For linearity assessment of myTAI_HEART_ DNA Fragmentation Assay amplifications for ALU115 and ALU247, gDNA was diluted in 0.1X TE to the following concentrations: 400, 200, 100, 50, 25, 12.5, 6.25, 3.13, 1.56 and 0.78 pg/μl. Each linearity sample was tested in duplicate wells per run, two runs per day for one day. The resulting ALU115 and ALU247 amplification measurements, quantitated against a standard curve in units of pg/μl as described in Materials and Methods, was plotted against the theoretical concentration and assessed for linearity according to CLSI EP06-A [92] (Fig 6). ALU115 and ALU247 results were individually assessed for linear, second and third order polynomial fits within JMP (SAS Institute., Cary, NC). For both, resulting p-values were <0.05 for linear fit and >0.05 for second and third order polynomial fits; R-squared values for linear fit were > 0.94 (ALU115) and >0.98 (ALU247), collectively indicating the results to be linear over the entire measured range of 0.78–400 pg/μl. (Table 8).

**Table 8:** Linearity fit values, myTAI_HEART_ DNA fragmentation assay.

Parameter	*ALU115 Assay*	*ALU247 Assay*
**Dynamic Range (pg/μl)**	0.78–400	0.78–400
**Linear Fit p-value**	< 0.0001	< 0.0001
**Linear Fit R-squared**	0.9436	0.9890
**Second Order Polynomial Fit p-value**	0.2498	0.2719
**Third Order Polynomial Fit p-value**	0.7215	0.6727

#### Interfering substances, myTAI_HEART_ DNA fragmentation assay

**Fig 7:**
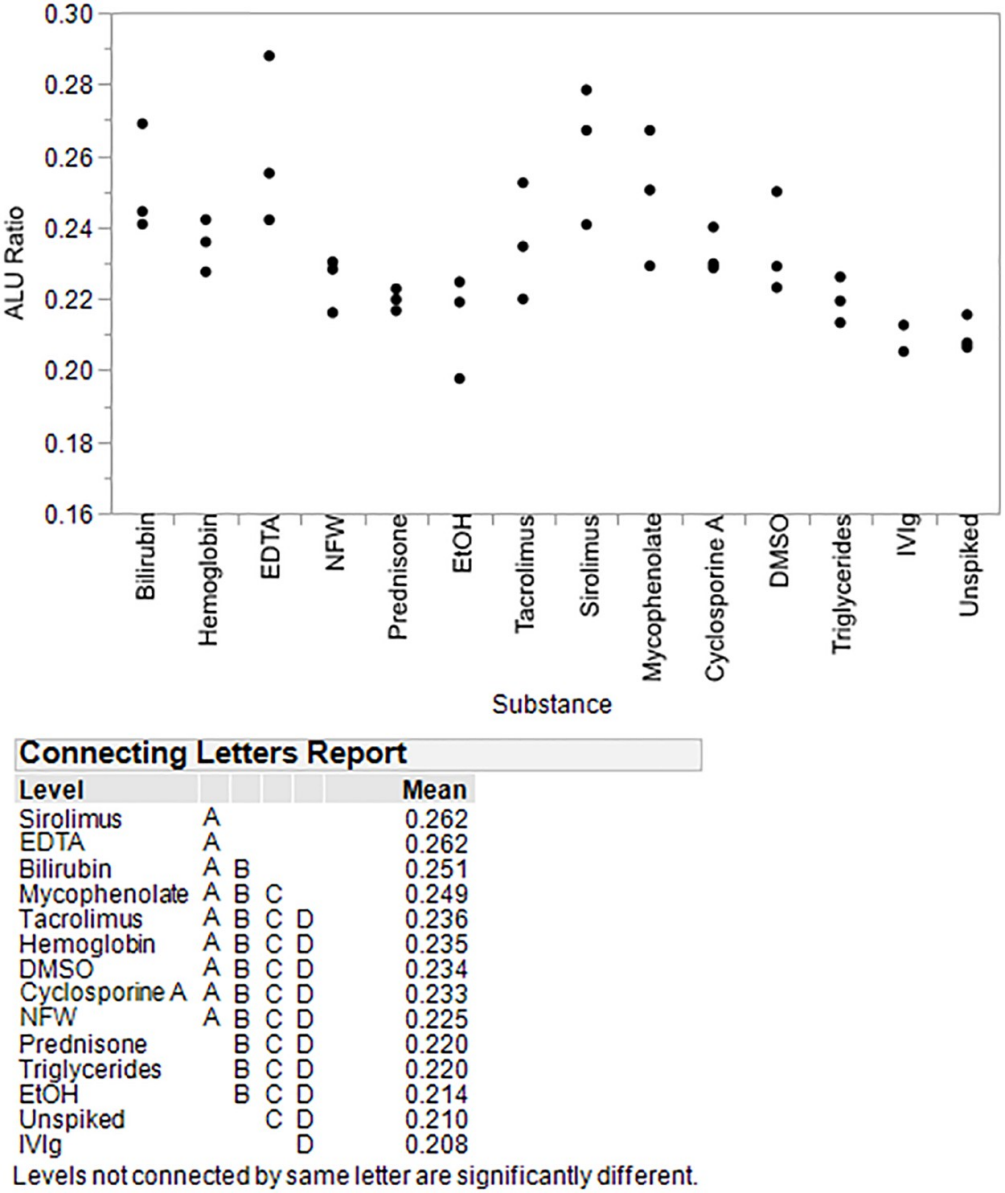
Interfering substance results at 25 ng/ml total cfDNA (TCF), DNA fragmentation assay, one-way analysis of *Alu* ratio with connecting letters report. At this TCF concentration, small, but statistically significant differences compared to the unspiked controls, but not compared to diluting solvent controls, were seen for Sirolimus, EDTA, and bilirubin. These findings, in concert with those at lower and higher cfDNA concentrations, indicate lack of clinically relevant effects of these substances on the DNA Fragmentation Assay (see text).

Effects of ten potentially interfering substances on the highly sensitive DNA fragmentation assay were individually assessed as described in Methods and Materials (see “Interfering Substance Assessment in Analytical Validations”) using three contrived human plasma samples prepared at three different clinically relevant TCF concentrations (2 ng/ml, 25 ng/ml, and 50 ng/ml) of variable *Alu* ratio. For each substance at each background cfDNA concentration, *Alu* ratio results were analyzed in JMP using the Tukey-Kramer HSD test following an ANOVA test to determine if the mean of the test case significantly differed from that of *an* “unspiked” control sample extracted and tested without added test substance or substance diluting solvent (water, DMSO, or ethanol). Results were also compared to those of a “solvent only” control sample spiked with a matched volume of the relevant diluting solvent without added substance. This allowed differentiation, within the bounds of the intrinsic variability of the ALU test, of any effect due to the substance itself versus any effect of the solvent required to dilute the substance for *in vitro* testing. “Solvent only” effects are not relevant to clinical test substance exposure. Importantly, for all tested substances, no statistically significant differences in *Alu* ratio of test samples compared to controls spiked only with diluting solvent were seen at any background cfDNA concentration. At 25 ng/ml TCF concentration, which falls well within the 95% confidence level clinical reference range for healthy heart transplant patients, small, but statistically significant differences compared to unspiked control were seen for Sirolimus, EDTA, and bilirubin, but not compared to the respective diluting solvent controls for these three substances (Fig 7). At very low TCF concentration occasionally seen clinically (2 ng/ml), a small, but statistically significant difference in *Alu* ratio compared to unspiked control was seen for hemoglobin (S4A Fig), but not compared to the solvent only control for hemoglobin (S4A Fig). No statistically significant effects of any of the 10 testing substances or their diluting solvents on *Alu* ratio were seen at cfDNA concentrations of 50 ng/ml cfDNA (S4B Fig). These findings indicate potential small *in vitro* effects of the solvents required to dilute test substances for interference testing, rather than clinically relevant effects of any of the tested substances themselves on the DNA Fragmentation Assay.

#### Detection of lysed leukocytes, myTAI_HEART_ DNA fragmentation assay, and effect on DF

**Fig 8:**
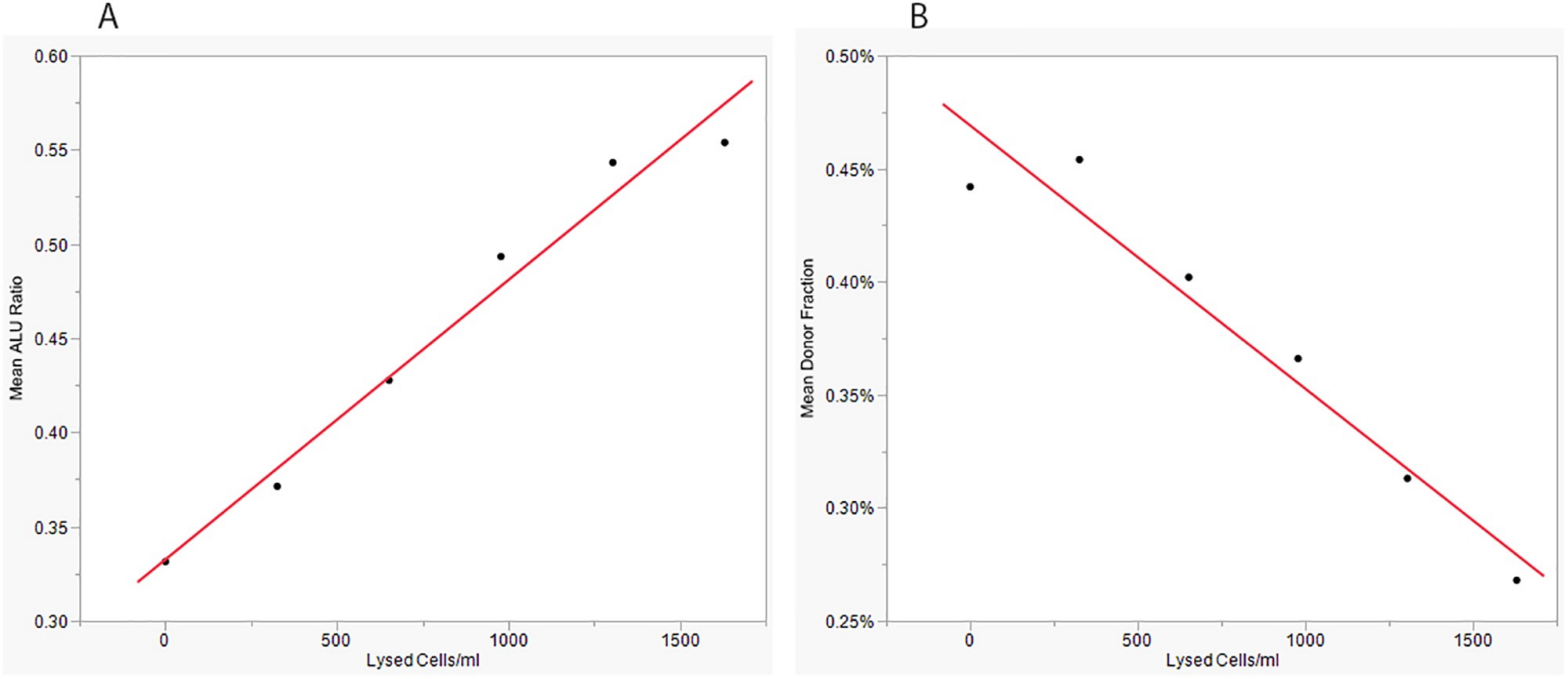
Quantitative effects of leukocyte lysis on Alu ratio (A) and DF (B).

The myTAI_HEART_ DNA Fragmentation Assay is designed to flag presence of excessive gDNA released from recipient leukocytes lysed during sample processing and shipping due to poor technique or extreme environmental exposures. Many published studies have demonstrated elevated TCF levels and/or increased proportions of long fragment DNA in plasma samples exposed to those conditions or delayed in separation of plasma from the cellular components of blood [95,100,108,116]. To assess effectiveness of the myTAI_HEART_ DNA Fragmentation Assay in detecting leukocyte lysis, we performed a leukocyte titration study using multiple 1.5 ml aliquots of a contrived sample prepared by spiking plasma from one healthy “recipient” blood donor sourced from blood bags provided by a commercial vendor with “donor” plasma from a second healthy subject. This yielded a theoretical DF of 0.4% in “post-transplant” plasma aliquots that were then spiked with specific numbers of leukocytes from the “recipient” donors buffy coat (enumerated by Cell Dyne cytometry), ranging from 0–2500 cells per 1.5 ml aliquot. After freezing at -80°C to lyse the leukocytes, samples were placed into the Method 2 myTAI_HEART_ workflow to determine DF and DNA fragmentation (ALU247/115) ratio per validated protocols. Results depicted graphically in Fig 8A and 8B indicate that, within the tested range, *Alu* ratio and DF changes are linear relative to quantitated addition of lysed cells. These results further show that the myTAI_HEART_ DNA Fragmentation Assay can detect elevations of *Alu* ratio by DNA derived from presence of as few as 300 lysed cells/ml of plasma, this representing roughly 0.003% of the leukocytes in whole blood from which that plasma is purified, based on normal reference range clinical leukocyte counts. Within the tested range of leukocyte contamination/lysis (300–1667 lysed cells /ml plasma), DF can drop from roughly 0.45% to as low as 0.275%. Even low levels of leukocyte lysis or contamination during sample processing have potential to shift DF from the high probability rejection range into the low probability range (producing a false negative result) if not monitored by DNA fragmentation analysis. Plasma samples most sensitive to risk for potential production of a false negative DF result due to leukocyte lysis are those with low TCF concentration and relatively low DF. Mathematical modeling to estimate that sensitivity is shown in S5 Fig.

**Fig 9:**
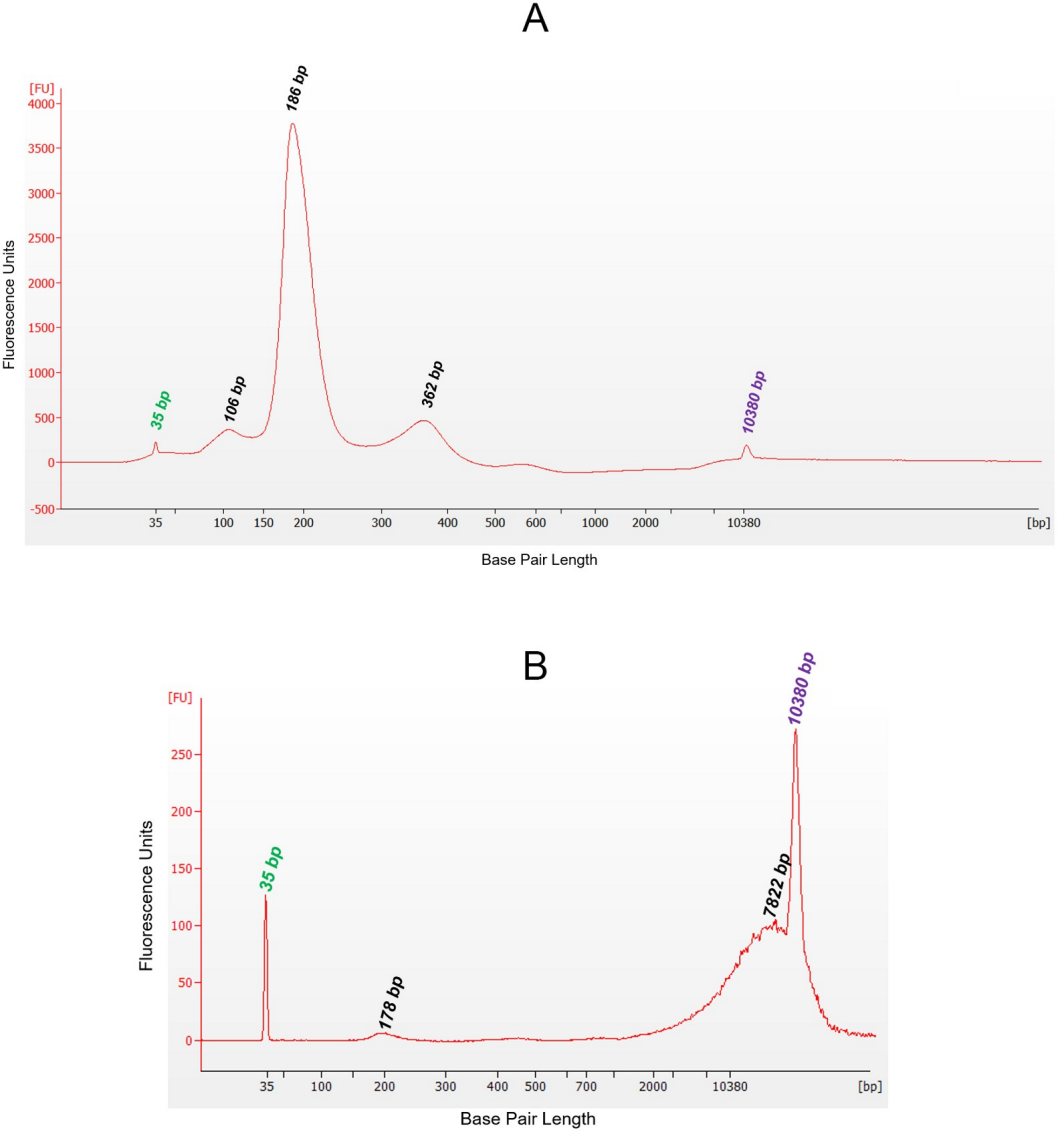
Bioanalyzer electropherograms of patient plasma cfDNA samples. (A) Patient sample collected and processed per TAI protocol shows predominant singlet and doublet apoptotic cfDNA peaks at 186 bp and 362 bp, respectively, without larger fragments produced by cellular lysis. (B) Human sample procured and processed by a commercial vendor with delayed centrifugation (> 24hrs) shows a small peak at 178 bp (probably apoptotic) and a large, broad peak centered at 7822 bp, consistent with origin from leukocyte lysis. In both figures, sharp peaks at 35 bp and 10380 bp are internal kit markers.

Capillary electrophoresis (*e*.*g*., Agilent Bioanalyzer) electropherograms, as previously shown for a contrived cfDNA reference sample in Fig 2, can be used for clinical quality assurance purposes to evaluate DNA fragmentation independently of qPCR in unusual patient plasma extracts with cfDNA concentration high enough to reach threshold sensitivity for this methodology (roughly 600 ng/ml) without over utilizing limited patient material. We used capillary electrophoresis to analyze the cfDNA fragmentation pattern of one such heart transplant patient (TCF concentration >6000 ng/ml and ALU115/247 ratio = 0.19), comparing the results of the *Alu* PCR-based myTAI_HEART_ DNA Fragmentation Assay to those of this independent method. The unusually high cfDNA level, with low DF, in this patient stemmed from acute renal tubular injury at time of myTAI_HEART_ blood sample collection following an episode of cardiac arrest and resuscitation prior to eventual recovery. The sample was processed through using the standard dual low-speed spin myTAI_HEART_ plasma preparation protocol, followed by automated extraction per Methods. It is informative to contrast the resultant electropherogram of the patient cfDNA extract collected by TAI protocol (Fig 9A) with one generated simultaneously for cfDNA extracted from plasma derived from a commercial normal donor blood lot shipped and received at TAI Diagnostics >24 hrs after collection (Fig 9B). It is clear from Fig 9A that even for cfDNA from this heart transplant patient with very significant *in vivo* non-cardiac cellular injury, the DNA fragmentation pattern is compatible with apoptosis as the primary mechanism of cfDNA origin. In contrast, the cfDNA population is largely long fragment (Fig 9B) in plasma commercially isolated and shipped without implementation of specific steps to avoid leukocyte lysis.

#### Carryover/cross-contamination, myTAI_HEART_ DNA fragmentation assay

Results of carryover/cross contamination analysis using contrived high positive samples generated at ~200 ng cfDNA/mL alongside negative samples (nuclease free water) in a checkboard pattern maintained throughout TECAN extraction and myTAI_HEART_ DNA Fragmentation analysis showed no evidence of carryover/cross contamination (S6 Fig). Positive and negative extraction controls assured run validity, and no data was removed from analysis. All negative samples tested measured at or below the LoD for the ALU115 and ALU247 measurements.

### Analytical performance characteristics: TCF extraction and quantification

#### Precision, TCF extraction and quantification

Table 9 reports the average, standard deviation, and percent CV (in ng cfDNA/mL plasma) across all extraction runs for five plasma samples spiked as per Materials and Methods with sheared DNA to span clinically relevant cfDNA concentrations of 2 ng/ml, 25 ng/ml, 50 ng/ml, 100 ng/ml, and 200 ng/ml. Extraction was performed in duplicate in each of 18 extraction runs using two lots of proprietary TAI extraction chemistry on two TECAN Freedom EVO 150 instruments by two technologists. The overall %CV was less than 15% across all concentrations. Results of ANOVA and F-tests did not find any of the individual testing variables described in Materials & Methods, including instrument, operator, run, and reagent lot differences, to exert effects of sufficient magnitude to impact TCF assay precision.

**Table 9:** TCF concentration: Precision averages, standard deviations, and %CV.

	2 ng/mL	25 ng/mL	50 ng/mL	100 ng/mL	200 ng/mL
**Average**	2.143	26.19	56.18	108.5	219.9
**St. Dev**.	0.311	2.93	6.93	14.7	29.5
**%CV**	14.5	11.2	12.3	13.5	13.4

#### LoB/LoD/LoQ, TCF extraction and quantification

In the LoB study of cfDNA extraction and quantification, which incorporated nine replicates of nuclease free water tested in each of six extraction and detection runs across four days using two reagent lots, there was no amplification in any sample. A non-normal distribution of the results requires the use of the nonparametric option when calculating the LoB. The LoB is 0 ng/mL. This predicts that whenever no DNA is present in a sample, TCF concentration results will be 0 ng/mL since no cross-reactivity of the primers or probe with themselves or any other component in the test system was detected in this study.

Numbers used to calculate LoD for TCF quantification according to CLSI EP17-A2, based upon testing of four distinct plasma samples in replicates of five in each of six extraction/detection runs across four days using two reagent lots, are detailed in Table 10.

**Table 10:** TCF quantification, limit of detection calculation.

Parameter	Reagent Lot 1	Reagent Lot 2
**SD**_**L**_	0.369	0.357
**n**_**i**_	15	15
**J**	4	4
**c**_**p**_	1.649	1.649
**L**	120	120
**LoB**	0	0
**LoD**	0.608 ng/mL	0.589 ng/mL

The study data exhibited a normal distribution; the parametric approach therefore was applied, assigning LoD for TCF quantification as 0.608ng/mL, the greater LoD of the two reagent lots.

Results and statistics used to determine the LOQ for TCF quantification are shown in Table 11, which provides the average TCF concentration, standard deviation, and %CV for each of five samples tested, by lot and as a pool. Every concentration tested generated a CV considerably less than 30%, and yielded a flat precision profile curve. The LOQ per CLSI guidelines was set at 3.03 ng/mL, the lowest concentration at which the % CV was confirmed to be less than 30%. This assigned LOQ is at the extreme low end of cfDNA concentrations seen in clinical samples using the RNaseP detection method and thus will not impact clinical utility. The predicted concentration at which the %CV would be 30% was less than the LoB. As a result, the LoQ per CLSI guidelines was set at 3.03 ng/mL, the lowest concentration at which the % CV was confirmed to be less than 30%.

**Table 11:** Limit of quantitation results, TCF.

TCF target concentration		Reagent Lot 1	Reagent Lot 2	Pooled
**2 ng/mL**	**Average**	2.97	3.09	3.03
	**St. Dev**.	0.46	0.31	0.39
	**%CV**	15.3	10.2	12.9
**5 ng/mL**	**Average**	6.92	6.90	6.91
	**St. Dev**.	0.73	0.71	0.71
	**%CV**	10.5	10.3	10.2
**10 ng/mL**	**Average**	13.62	14.08	13.83
	**St. Dev**.	1.89	1.81	1.84
	**%CV**	13.9	12.9	13.3
**15 ng/mL**	**Average**	20.28	21.35	20.80
	**St. Dev**.	2.31	2.36	2.36
	**%CV**	11.4	11.1	11.3
**20 ng/mL**	**Average**	27.37	27.74	27.55
	**St. Dev**.	3.23	2.18	2.73
	**%CV**	11.8	7.8	9.9

#### Linearity, TCF extraction and quantification

**Fig 10:**
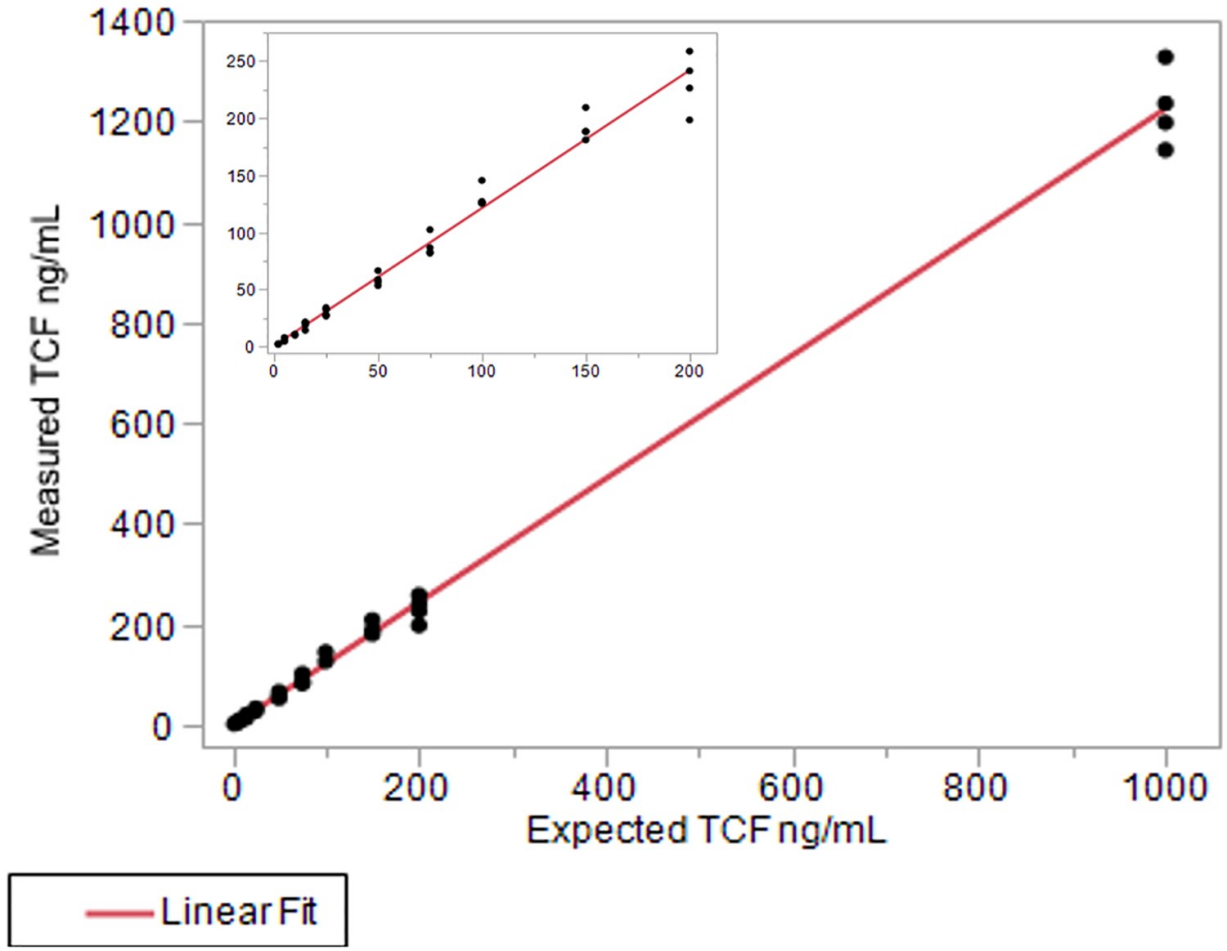
TCF linearity 2–1,000 ng/mL. The TCF assay is linear from 2–1,000 ng cfDNA/mL plasma. The adjusted linear fit equation is ng/mL (y) = -0.455701 + 1.2255499*Expected ng/mL(x).

Samples that generated RNase P values above the highest standard curve point were diluted and retested such that they fell within the standard curve. The resulting quantifications were then multiplied by the appropriate dilution factor to back-calculate the starting concentration. The linear, second order and third order fits were assessed across the entire data set (2–6,000 ng/mL). In this range, there were significant fits for both the linear and second order polynomial. The second order polynomial fit is not statistically significant when the range is reduced to 2–1,000 ng/mL. Fig 10 plots the linear range.

#### Interfering substances, TCF extraction and quantification

Effects of ten potentially interfering substances on TCF determination were assessed as described in Methods and Materials (see “Interfering Substance Assessment in Analytical Validations”) in parallel with and without deviation from these assessments performed for the myTAI_HEART_ DNA Fragmentation Assay previously described. For the included reconstructed plasma samples (TCF concentrations of 25 ng/mL and 50 ng/mL), results of Tukey-Kramer analysis indicated that samples spiked with each tested substance were statistically in the same group as those spiked only with the diluting solvents (S7 Fig). Therefore, no test substance caused interference.

#### Carryover/cross-contamination, TCF extraction and quantification

Carryover/cross contamination was assessed in parallel with and without deviation from the checkerboard-style testing of positive and negative samples applied to the DNA Fragmentation Assay above. No data was removed from analysis. All negative samples remained negative, indicating the extraction and quantification workflow was not affected by carryover/cross contamination (S8 Fig).

#### Determination of a TCF reference range in a population of asymptomatic heart transplant recipients

**Fig 11:**
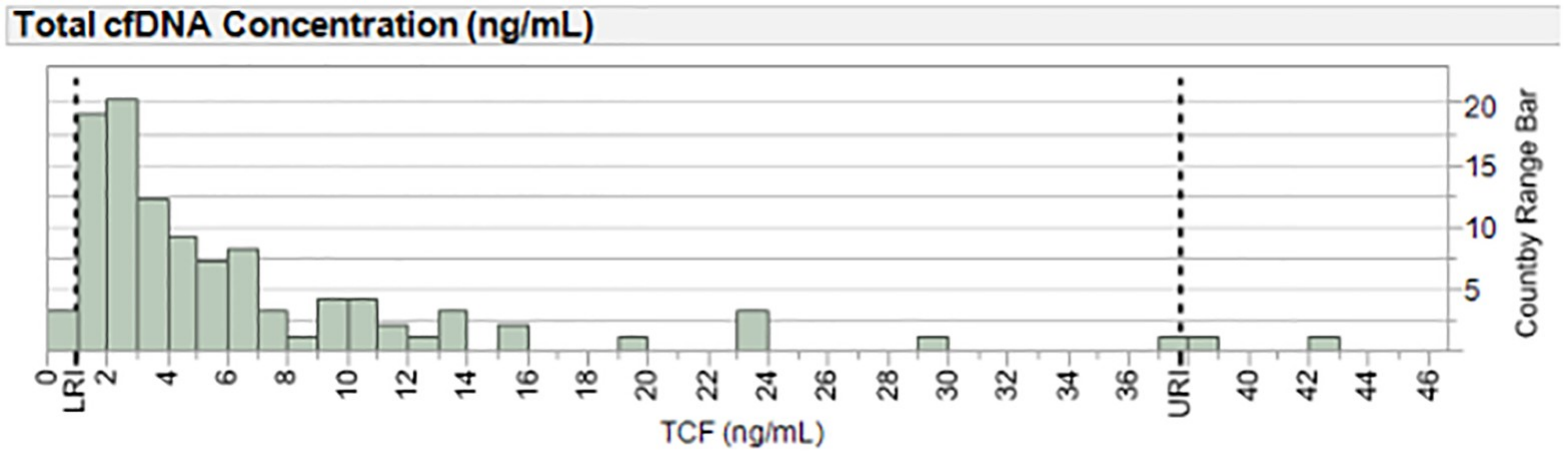
Distribution of TCF concentration within asymptomatic healthy heart transplant recipients (264 samples from 106 subjects).

**Fig 12:**
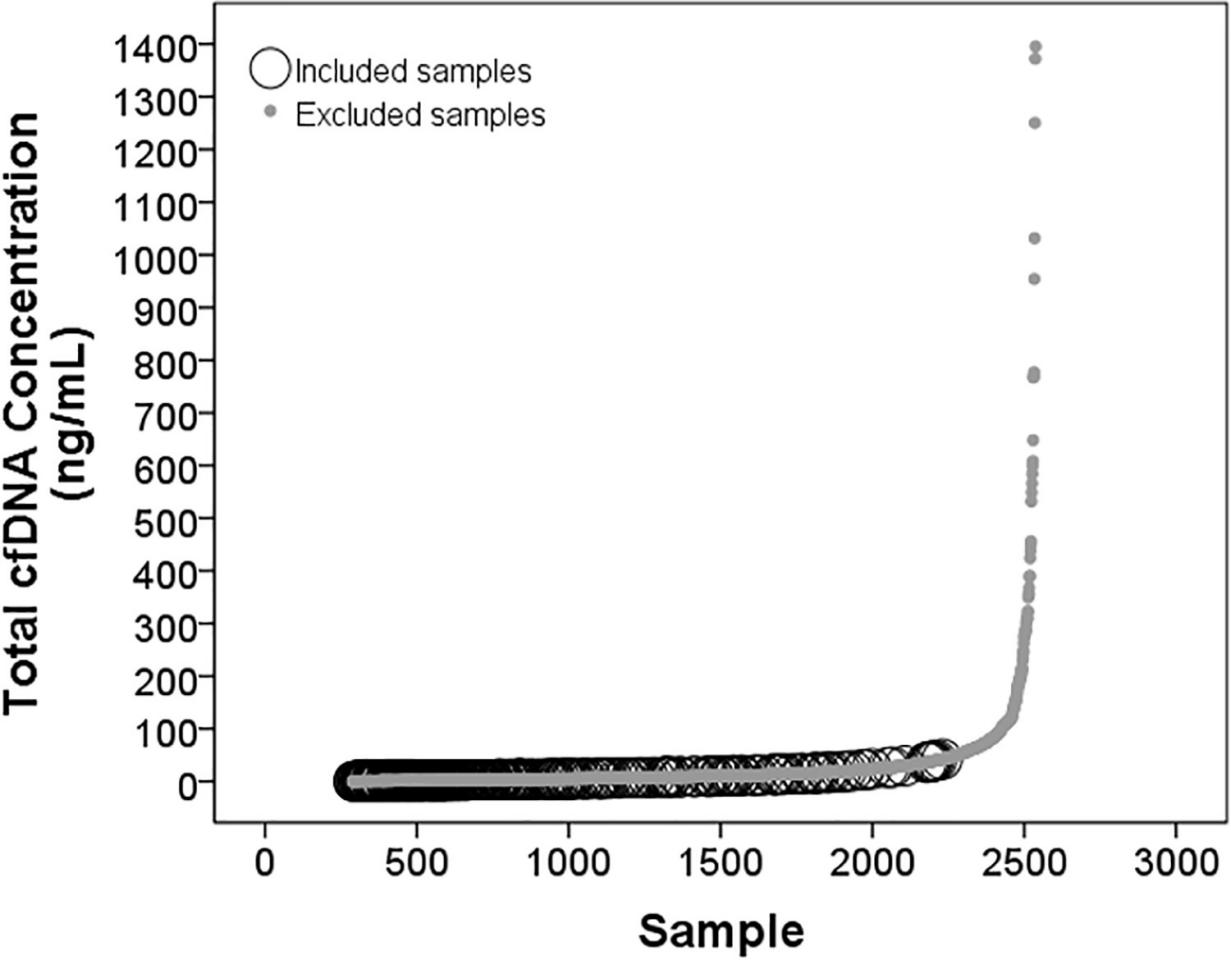
Distribution of TCF concentration in included (healthy) and excluded (potentially unhealthy) cohorts of the heart transplant recipient population. Open circles represent the 264 “healthy” samples after exclusions; grey dots represent excluded samples from potentially “unhealthy” subjects. Samples are linearly arranged along the x-axis in order of increasing TCF (total cfDNA) concentration.

Using TCF concentration measurements determined for 264 samples from 106 asymptomatic heart transplant patients from Aim 1 of the DTRT study [89,90], the 95% reference interval was established as 0.91–37.70 ng/ml (mean 6.70 ng/ml) using the nonparametric method. Distribution of TCF levels in this population (averaged for each subject) are shown in Fig 11. Differential distributions of TCF levels in with the included (healthy) and excluded (unhealthy) populations are shown in Fig 12.

### myTAI_HEART_ basic genotyping analytical performance characteristics

Reproducibility/precision testing of the basic-genotyping (bGT) test was performed using six separate patient whole blood samples in six different extraction/library amplification runs performed by two different technologists over a span of seven days. Across all samples, runs, and targets (6 x 6 x 95 = 3120 individual amplifications), 3123/3420 (91.3%) generated concordant calls, 293/3420 (8.6%) did not generate a call, and 4/3420 (0.12%) generated discordant calls. Overall, all samples generated concordant genotype calls across all runs with exception of a single target in one sample and another single target in another sample. Accuracy of myTAI_HEART_ bGT results was verified in comparison to paired Illumina sequencing of 14 amplified DNA libraries across four control samples. On average, 82/94 SNPs were called with confidence on both platforms, and in every instance the genotype call was equal.

### myTAI_HEART_ quantitative genotyping analytical performance characteristics

#### LoB/LoD/LoQ, quantitative genotyping (DF determination)

**Fig 13:**
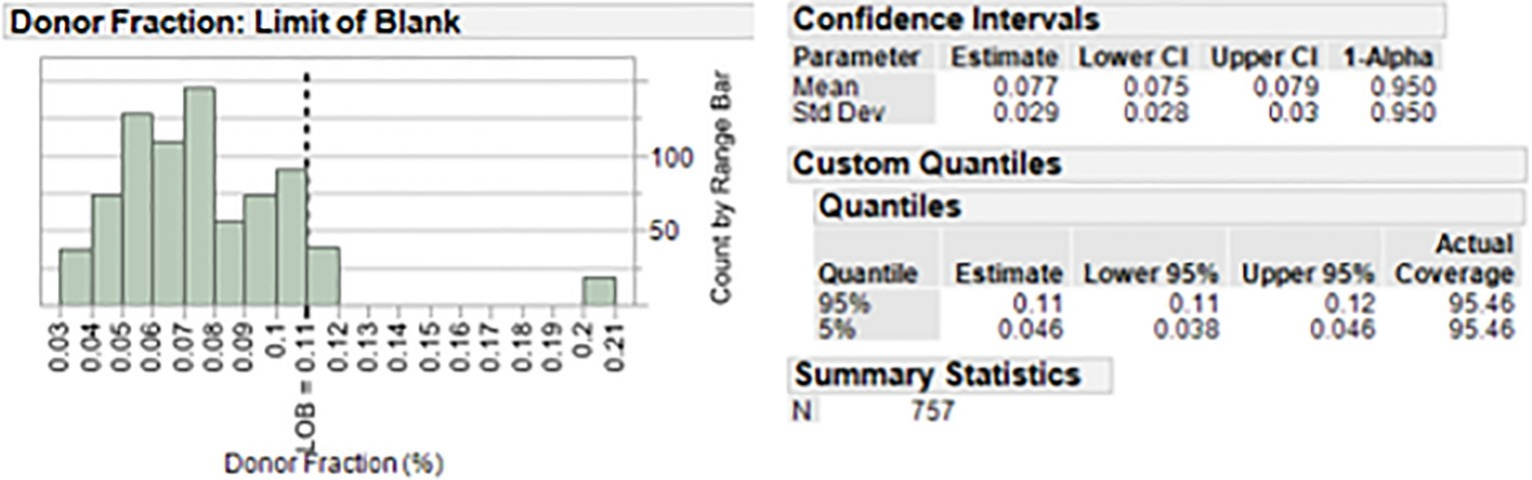
Limit of blank distribution, DF determination by quantitative genotyping. LoB = 0.110% using the classical nonparametric approach applied to 757 samples (see text). Note that no estimated “system noise” has been subtracted from the LoB, yielding a pure LoB reference value for DF determination.

Analytical performance characteristics of the critical quantitative genotyping (qGT) portion of the assay that determines post-transplant DF were evaluated per CLSI EP17-A2 Evaluation of Detection Capability for Clinical Laboratory Management Measurement Procedures [91] as described in Materials and Methods. LoB for DF quantification was determined using the classical nonparametric approach per that document and as described in Materials and Methods. A total of 757 measurements were used in the final LoB analysis, and the calculated rank position was 720 (rank position = 0.5+757*0.95). The donor fraction at that position, defined as the LoB, was 0.110% (Fig 13).

LoD for DF quantification was determined using the equation LoD = LoB + Cp*SDI for each of 9 distinct reconstructed samples made at a theoretical DF of 0.1% in triplicate, each amplified in triplicate at an input of 15 ng DNA on each of three days on each day using a different mastermix reagent lot. Two sample replicates failing library amplification were removed from the analysis. LoDs for the three different reagent lots were 0.165%, 0.149%, and 0.153% (Table 12). The overall LoD, defined as the greatest value across all three reagent lots, was thus determined to be 0.165%.

**Table 12:** Limit of detection calculations, quantitative genotyping (DF).

Mastermix Reagent Lot	Date	LoB	Cp	SD_i_	LoD
**Lot 1**	2/23/18	0.110	1.67	0.0330%	0.165%
**Lot 2**	2/24/18	0.110	1.67	0.0236%	0.149%
**Lot 3**	2/25/18	0.110	1.67	0.0258%	0.153%

LoQ for DF quantification was determined for nine distinct reconstructed samples made at each of three theoretical donor fractions (0.1%, 0.2%, and 0.3%), each amplified in triplicate libraries then quantified for DF. Each of these library runs was tested across multiple days with distinct reagent lots. For each reconstructed sample, DF data from the triplicate library runs was pooled to generate the %CV for each of the nine reconstruction samples (Table 13). The average %CV for each DF fraction level was plotted along with the best fit line, generating a precision profile curve used to estimate the level at which the precision of the assay was ≤ 20%CV. Using the calculated fit equation, the minimum DF level (%) at which the precision of the assay is ≤ 20% CV is 0.108% (S9 Fig). By definition, the LoQ must be greater than or equal to the LoD; therefore, the LoQ is equal to the LoD and is thus 0.125%.

**Table 13:** %CV of DF measurement for reconstructed samples at three DF levels.

	0.1% DF	0.2% DF	0.3% DF
**Sample 1**	14.58%	10.94%	12.65%
**Sample 2**	19.38%	7.33%	4.97%
**Sample 3**	14.09%	9.82%	21.25%
**Sample 4**	22.13%	13.08%	6.19%
**Sample 5**	9.00%	13.11%	11.81%
**Sample 6**	13.66%	17.36%	14.13%
**Sample 7**	28.24%	9.93%	12.10%
**Sample 8**	15.46%	22.14%	7.03%
**Sample 9**	13.91%	11.30%	14.83%
**Average (n = 9)**	**16.72%**	**12.78%**	**11.66%**

#### Precision and accuracy, quantitative genotyping (DF determination)

Precision results for DF determination across the dynamic range of the myTAI_HEART_ assay relied upon use of three reconstruction control samples generated at low (~0.20%), medium (~1.0%), and high (~10%) donor fractions. These were aliquoted into single use portions that were tested as a single replicate up to two times a day over 31 days for a total of 46 replicates per sample, tested by multiple operators across several reagent lots and equipment lines as described in Materials and Methods. Input DNA mass was 15 ng for all samples for a total of 46 replicates per sample. The %CV within run date and across all samples was determined for each sample (Table 14). All sample replicates that failed QC were removed from the analysis.

**Table 14:** Precision of DF determination across days, operators, reagent lots, and equipment lines for three reconstructed samples (low, medium, and high DF level).

Reconstructed Sample	Measured DF (Mean)	Standard Deviation	%CV
**Low DF (0.2%)**	0.18%	0.04%	22.92%
**Medium DF (1.0%)**	1.19%	0.15%	12.31%
**High DF (10%)**	10.5%	0.779%	7.39%

Accuracy of myTAI_HEART_ DF determination was verified for six amplified DNA libraries across the three reconstruction control samples of Table 14 by comparing their myTAI_HEART_ DF results to DF results obtained by sequencing on an Illumina MiSeq using standard PCR amplicon sequencing protocols and paired 150 bp fragments. Z scores were calculated using historical means and standard deviations of myTAI_HEART_ control sample data and empirical values determined for the six sequenced libraries. For each sample, median absolute difference (Z score) of myTAI_HEART_ DF compared to sequencing DF was less than one standard deviation, indicating good concordance between methodologies.

#### Linearity, quantitative genotyping (DF determination)

**Fig 14:**
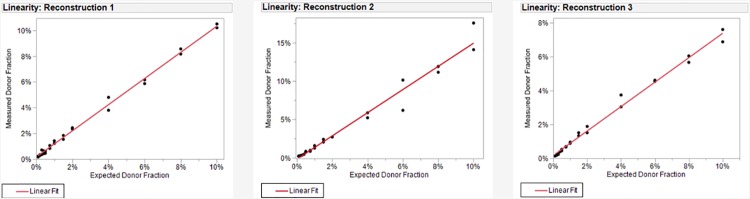
Linearity of DF in three blood lot reconstruction series.

Linearity results for DF determination, as described in Materials and Methods and performed in accordance with CLSI EP06-A Evaluation of the Linearity of Quantitative Measurement Procedures, were based on use of three reconstruction series manufactured to represent DF’s of 0.10, 0.20, 0.30, 0.40, 0.50, 0.75, 1.00, 1.50, 2.00, 4.00, 6.00, 8.00, and 10.00%. These were all tested in duplicate and processed in the same run with a single lot of reagents. One sample in one series failed RNase P quantification, indicating failed library amplification, and was removed from analysis. Fig 14 depicts measured DF versus expected DF based for the three reconstruction series. The pooled reconstruction series were assessed for linear, second and third order polynomial fits within JMP, yielding a linear fit p-value of <0.0001 in the dynamic range of 1–10% DF. The linear fit equation was y = 1.08 (±.05) x + 0. Second and third order polynomial fit p-values were not statistically significant (0.6691 and 0.5635, respectively).

#### Interfering substances, quantitative genotyping (DF determination)

**Fig 15:**
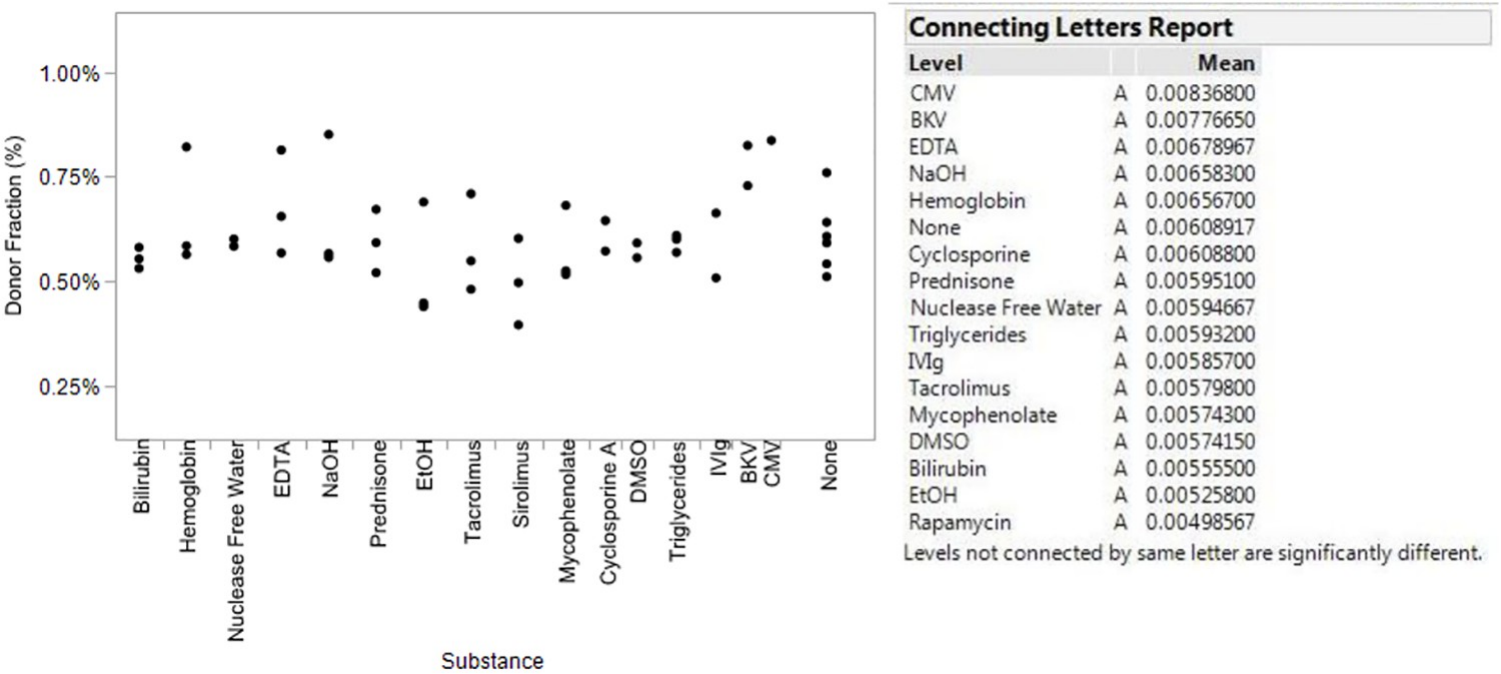
Interfering substance testing, quantitative genotyping, 0.6% DF reconstruction.

Effects of 10 clinically relevant, potentially interfering substances and two viruses (CMV and BKV) on determination of DF by qGT were evaluated individually, in triplicate, as described in Materials and Methods for two unique contrived plasma samples. These were prepared by mixing plasma from two individuals in different proportions to simulate plasma from two transplant patients with donor fractions of ~0.6% and ~1.0%. For each contrived sample, un-spiked aliquots and aliquots spiked only by solvents used to dilute each tested substance were run alongside as comparators. Two replicates (one spiked with Cyclosporine and one spiked with DMSO) failed library amplification and were removed from analysis. Tukey-Kramer HSD analysis indicated that the mean DF result of all substance-spiked replicates was statistically equivalent to the mean DF result of their paired samples spiked with solvent only as well as that of the un-spiked sample. All potentially interfering substances were also equivalent to each other. The results of interfering substance testing at both DF levels showed that prescription drugs, endogenous substances, and pathogens common in the heart transplant population do not significantly affect DF results, shown for the 0.6% DF reconstruction in Fig 15.

#### Carryover/cross-contamination, quantitative genotyping (DF determination)

**Fig 16:**
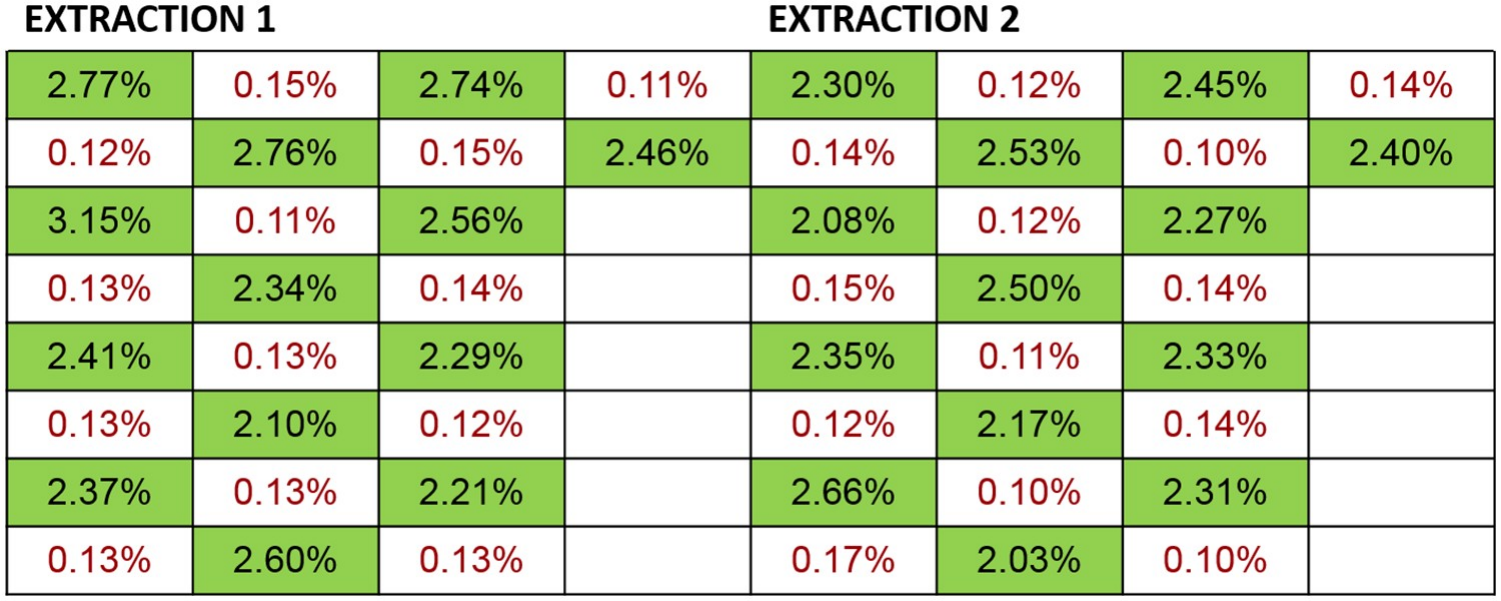
DF (%) results, TECAN extractions 1 and 2, carryover/cross-contamination testing. Relative sample positions were maintained from extraction through the entire myTAI_HEART_ workflow (extraction through qGT). Green positions = high DF samples; white positions = low DF samples.

Potential carryover/crossover contamination during extraction and all downstream workflows leading to final determination of a valid DF was evaluated as described in Materials and Methods using contrived plasma samples (see Reference Materials) generated at low DF (~0.2%, below cut-off) and high DF (~2.0%, high above cut-off). These were extracted on duplicate TECAN runs in a checkboard pattern that was maintained throughout subsequent processing through the full myTAI_HEART_ qGT workflow to produce DF results (Fig 16). All samples met QC criteria; none were removed from analysis. All low DF samples remained low when processed alongside high positive samples, indicating the testing system is not subject to carryover/cross-contamination.

### Clinical performance characteristics of myTAI_HEART_ DF as an index of low vs increased risk of moderate to severe acute cellular rejection

The intended clinical use of the myTAI_HEART_ assay is to aid in identification of heart transplant recipients who have a low versus increased risk of moderate/severe ACR (ISHLT 2005 grade 2R or higher) at time of testing in conjunction with standard clinical assessments. The final clinical validation dataset selected according to the defined exclusion and inclusion criteria described in Methods included 158 matched pairs of endomyocardial biopsy-plasma samples collected from 76 heart transplant recipients, both pediatric and adult (41% female, 59% male). The age range of these patients was 0.28–28.00 years (mean 10.85 years), including five patients aged less than 1 year, 16 from 1–5 years, 20 from 5–10 years, 13 from 10–15 years, 10 from 15–20 years, and 12 from 20–28 years. Of the 158 biopsies, 148 were asymptomatic surveillance biopsies. Racial/ethnic composition of the 76 subjects was 3% Asian, 9% Black or African American, 84% white, 1% Native American, 3% unreported. Using this dataset, a DF cutoff of 0.32% was selected to maximize the negative predictive value (NPV) for grade 2R or higher ACR by establishing a cutoff for grades 1R or higher (mild, moderate, and severe rejection) vs grade 0R (no rejection). DF increased across rejection grades: the median DF in 0R (n = 134) was 0.12% (IQR 0.09–0.23%), in 1R (n = 21) was 0.84% (IQR 0.21–4.64%), and in 2R (n = 3) was 1.04% (IQR 0.85–3.13%). Note that these DF mean values, and their interquartile ranges, are slightly different than previously reported for this sample set [66] due to application of the definitive, clinically validated myTAI_HEART_ bioinformatics algorithm here and a developmental version previously.

**Fig 17:**
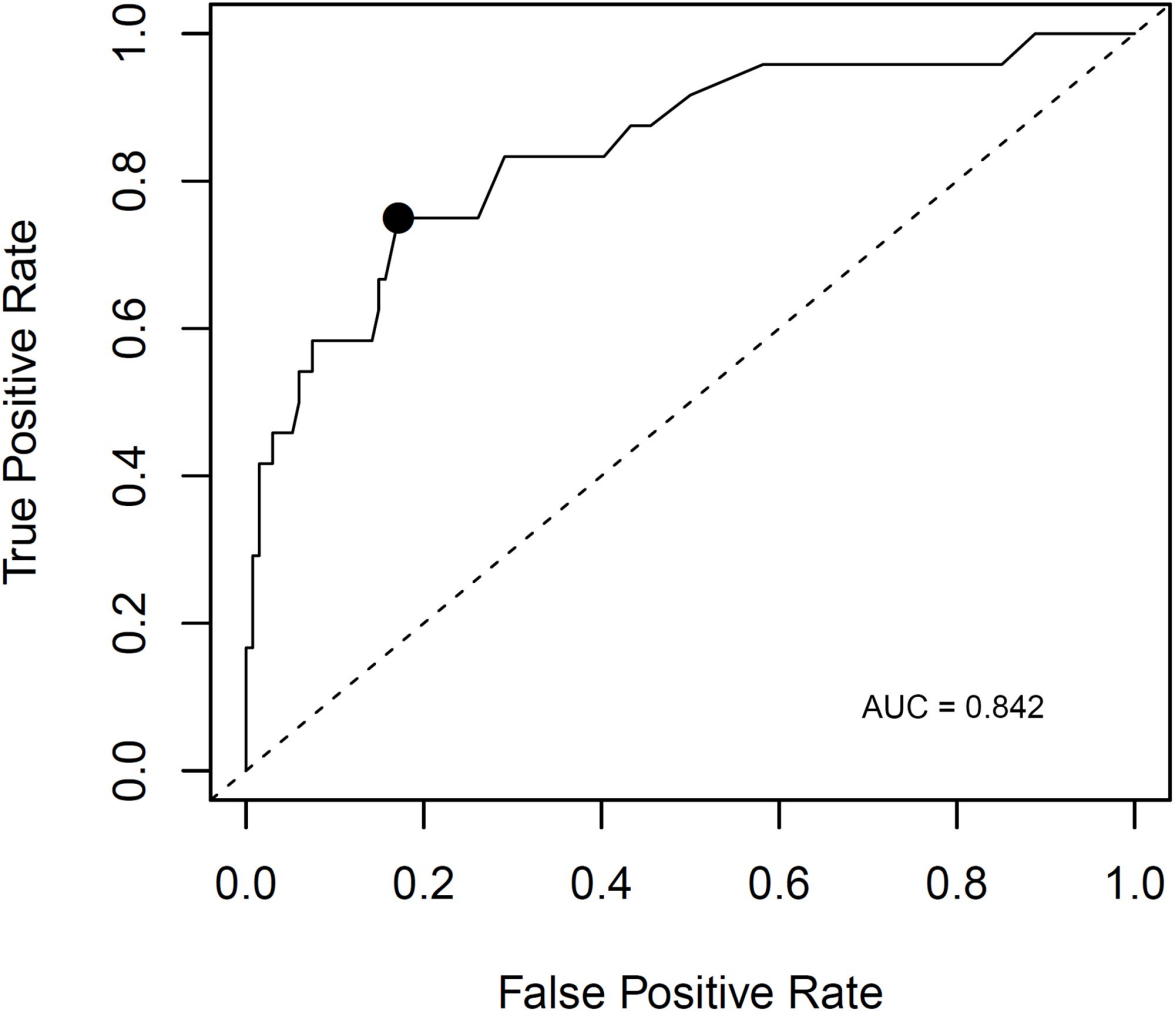
Receiver operating characteristic (ROC) curve, ACR 0R versus ACR 1R+2R+3R. Area under the Curve (AUC) was a robust 0.842. Using the DF cutoff of 0.32%, NPV for grade 2R or higher ACR, the intended use of the myTAI_HEART_ assay, was 100.00% for grade 2R or higher ACR, with 100.00% sensitivity and 75.48% specificity.

Based upon ACR grade as defined by the 2004 ISHLT classification, Receiver Operating Characteristic (ROC) analysis was performed to evaluate diagnostic accuracy across all possible cutoffs. To maximize diagnostic accuracy, Youden’s Index was used to select the optimal cutoff, found to correspond to a DF value of 0.32%. Using this cutoff, clinical performance characteristics of the assay included an NPV of 100.00% for grade 2R or higher ACR, with 100.00% sensitivity and 75.48% specificity; Area under the Curve (AUC) for this analysis was 0.842, indicative of robust ability of the DF assay to rule out 2R or greater ACR for DF values less than 0.32% Fig 17). In addition, when using the 0.32% DF cut-off value, the assay demonstrated 94.87% NPV and 43.90% PPV for grade 1R versus grade 0R, emphasizing the sensitivity of the DF determination to detect many cases of mild ACR. Sensitivity for ACR 1R may also reflect the fact that patients with recurrent rejection classified as 1R according to the 2004 ISHLT classification, which combines grades 1A, 1B, and 2 of the 1990 classification, have decreased freedom from late ACR and poorer long-term outcomes when of 1990 ISHLT 1B/2 histology [117,118]. Also of note, within this validation sample set, presence of coronary artery vasculopathy (CAV) correlated with increased DF [66]. The exquisite sensitivity and rapid response of the test to myocardial injury is demonstrated by a median 7.5 fold increase in cfDNA genomic equivalents/ml across all patient ages and weights within 15 minutes post-endomyocardial biopsy, this increase significantly higher in smaller and younger patients [119].

We found no statistically significant correlation of DF with age across the broad developmental age range of our study. Predictably, there was some variability in distribution of cases of significant rejection across age subgroups, since each subgroup was of relatively small N and influenced by intrinsic weighting of shorter interval between transplantation and sample collection in younger age groups. There was no evidence to suggest that age differences would challenge the low, highly conservative myTAI_HEART_ DF cut-off threshold for increased risk of moderate to severe ACR. To test this observation in a larger population of heart transplant patients and samples, we correlated myTAI_HEART_-determined DF values patient age at time of sample collection from three combined QC-passed study cohorts which included the current clinical validation study set, the AIM1 cohort from the previously cited DTRT study [89], and the TAI Diagnostics registry. A plot (S10 Fig) of patient age at time of sample collection versus DF shows for this combined cohort showed no trend between pediatric and adult values (linear model age vs DF p = 0.29 or p = 0.52 if controlling for known ACR grading). Patient-specific health outcomes confound categorical analysis (all samples associated with biopsy diagnoses of ACR>1, for instance, were from patients <19 years of age), driving a difference in means all pediatric vs adult p = 0.03). Data includes study subjects (N = 180) and clinical registry records (N = 96).

## Conclusions

The intended use of this highly sensitive, clinically accredited PCR-based assay for selective damage to a donor heart is designed conservatively to stratify low versus increased probability of moderate to severe ACR in heart transplant recipients with 100% NPV based on a DF cut-off value. The analytical and clinical validation data reported herein strongly supports its validity in doing so. This test is validated for clinical diagnostic use in heart transplant recipients who are 2 months of age or older and as early as 8 days post-transplant. It significantly expands the window of opportunity for noninvasive transplant rejection assessment to infants and young children and to all recipients, adult or pediatric, into the critical early post-transplant period in which rejection is most common.

It is important to realize that cfDNA DF elevation within the reportable range of this highly sensitive assay can also be caused by other forms of selective cellular injury to the donor heart such as ACR 1R, acute antibody-mediated rejection (AMR), and CAV. Therefore, as with any single test, myTAI_HEART_ results require correlation with other clinical indicators to guide patient care interventions. Although the most compelling value of the test is a negative myTAI_HEART_ test result (DF < 0.32%), which with its 100% NPV for 2R/3R ACR confidently assures absence of significant acute rejection, rare false negative results are possible due to unusual clinical conditions. Heart transplant recipients with a negative result should continue to be monitored according to standard clinical care. Heart transplant recipients with a positive result should receive further evaluations, guided by clinical judgement and results of other testing modalities, to determine the cause. “Biopsy negative” rejection is a distinct possibility. In all instances of cfDNA analysis, regardless of target or methodology, it is essential and achievable to address the universal concern for potential false negatives caused by artefactual contamination of plasma with leukocyte gDNA. Such contamination can originate from leukocytes not removed completely or lysed during improper sample processing, or more rarely through *in vivo* acute leukocyte injury from concurrent non-cardiac biological insult or therapeutic intervention (e.g., cytotoxic drug infusion). The myTAI_HEART_ test addresses these challenges in part by instituting a simple, rapid sample shipping and processing protocol easily performed in collecting clinical laboratories that assures high plasma quality at the point of collection. It also employs a clinically validated DNA fragmentation “safety net” assay at the point of testing that verifies absence of an unacceptable level of long fragment, non-apoptotic, cfDNA which would be indicative of problematic leukocyte contamination or lysis. It is also important to acknowledge that patients recently treated for rejection may have variably affected DF’s. The latter variability is not yet well investigated, but is avoided by the 28-day post-treatment warning of the myTAI_HEART_ assay. Accrued clinical experience with use of the test will likely narrow this post-treatment interval. Trending through relatively frequent longitudinal serial testing may be helpful in some patients.

In real-life clinical application of myTAI_HEART_ testing, a number of scenarios have rapidly surfaced that particularly benefit from myTAI_HEART_ cfDNA DF analysis, for example:

- Patients with limited vascular access precluding or complicating biopsy
- Stable patients past the first post-transplant year who would benefit from reduced biopsy incidence
- Patients shifted to monotherapy, requiring more frequent monitoring
- Patents too ill for anesthesia and biopsy
- Patients needing closer non-invasive serial follow-up to a recent positive biopsy
- Patients with clinical suspicion of rejection, but negative biopsy
- Patients with shortness of breath of respiratory versus cardiac origin

Additional and refined applications will presumably develop over time as clinical experience in daily practice with cfDNA testing accrues. This noninvasive testing option is particularly critical in meeting unmet clinical needs for infants and young children not eligible for other noninvasive interventions, but is also applicable to adolescents and adults.

To be clear, the myTAI_HEART_ test is not intended to completely replace EMB, which can provide uniquely advantageous morphological, immunohistochemical, and molecular diagnostic information, but rather to complement and/or reduce the incidence of invasive biopsy while providing its own game-changing advantages of sampling uniformity, objectivity, cost-effectiveness, and safety. The validation studies presented here comprehensively document the analytical validity and clinical performance characteristics of the myTAI_HEART_ test, including its DF and TCF determinations and accompanying DNA Fragmentation quality control analysis. These, together, support reliable clinical utility in conservative stratification of probability of moderate to severe ACR, providing an immediate alternative to invasive endomyocardial biopsy for pediatric and adult patients with contraindications to biopsy as well as for all who would benefit from more frequent monitoring. A*dditional prospective studies evaluating long-term outcomes across a variety of clinical scenarios using the test versus biopsy alone are needed to establish its clinical utility further in reducing the incidence of need for invasive endomyocardial biopsy and to evaluate its performance in following response to therapy*. This is a precise, quantitative test and has potential in the future to provide quantitative measures of transplant health beyond the current binary risk stratification. Larger scale clinical studies over coming years will more fully enlighten the myriad potential clinical utilities and limitations of cfDNA analysis in the care of heart transplant patients.

## Supporting information

S1 Fig Contrived plasma reference samples of targeted TCF concentration and DF. (A) At three targeted DF levels of contrived reference materials, the expected TCF concentration of 20 ng/ml was closely approximated, analyzed by the All Pairs, Tukey HSD test (also called Tukey-Kramer test). (B) Correlation of measured and expected DF, each error bar constructed using one standard error from the mean.
(TIF)

S2 Fig LoB measurement distributions, ALU115. LoB, calculated according to the CLSI nonparametric option [91] and denoted by the dashed vertical line is 0.014 pg/μl.
(TIF)

S3 Fig LoB measurement distributions, ALU 247. LoB, calculated according to the CLSI nonparametric option [91] and denoted by the dashed vertical line is 0.006 pg/μl.
(TIF)

S4 Fig DNA fragmentation assay interfering substance testing. Testing results at 2ng/ml TCF (A) and at 50 ng/ml TCF (B). For results at 25 ng/ml TCF see Text, Fig 7.
(TIF)

S5 Fig Mathematical modeling of theoretical DF reduction due to excess leukocyte gDNA. (TIF)

S6 Fig DNA fragmentation assay crossover study. (TIF)

S7 Fig TCF interference testing results. (TIF)

S8 Fig TCF concentration cross-over study. (TIF)

S9 Fig LoQ precision profile Curve, DF determination. The average %CV at each DF is shown, along with the calculated best-fit line. The DF level along that line at which precision of the assay was ≤ 20%CV, is 0.108%, indicated by the vertical marker.
(TIF)

S10 Fig Patient age distribution of DF level over three combined study cohorts. Data includes study subjects (N = 180) and clinical registry records (N = 96). See text for statistical interpretation.
(TIF)

S1 Table Interference testing substances and concentrations. (DOCX)
