# Supplementary figures and images for: Cell-free DNA donor fraction analysis in pediatric and adult heart transplant patients by multiplexed allele-specific quantitative PCR: Validation of a rapid and highly sensitive clinical test for stratification of rejection probability
Source: PLoS One. 2020 Jan 13;15(1):e0227385. doi: 10.1371/journal.pone.0227385 (PMC6957190; doi:10.1371/journal.pone.0227385)

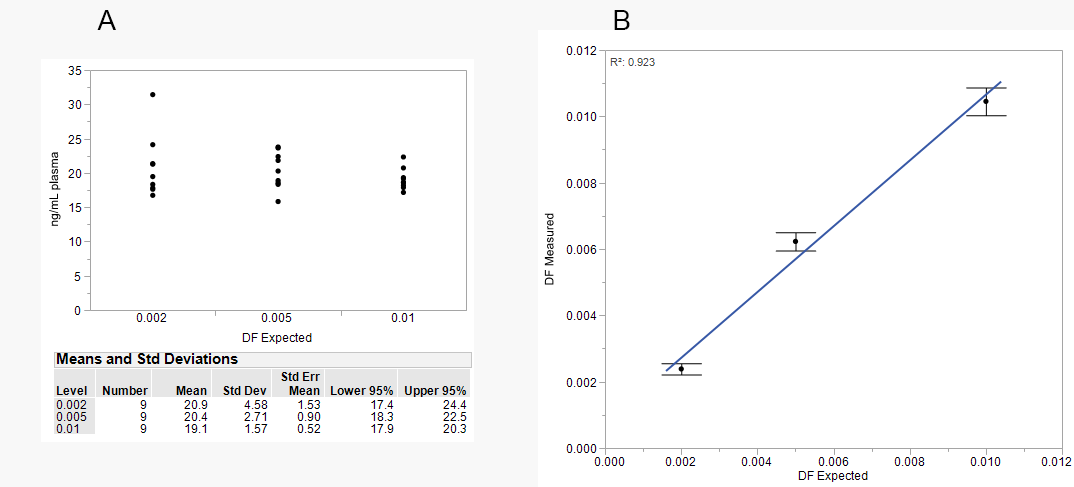

Supplement: S1 Fig — (A) At three targeted DF levels of contrived reference materials, the expected TCF concentration of 20 ng/ml was closely approximated, analyzed by the All Pairs, Tukey HSD test (also called Tukey-Kramer test). (B) Correlation of measured and expected DF, each error bar constructed using one standard error from the mean. (TIF) [file pone.0227385.s001.tif]

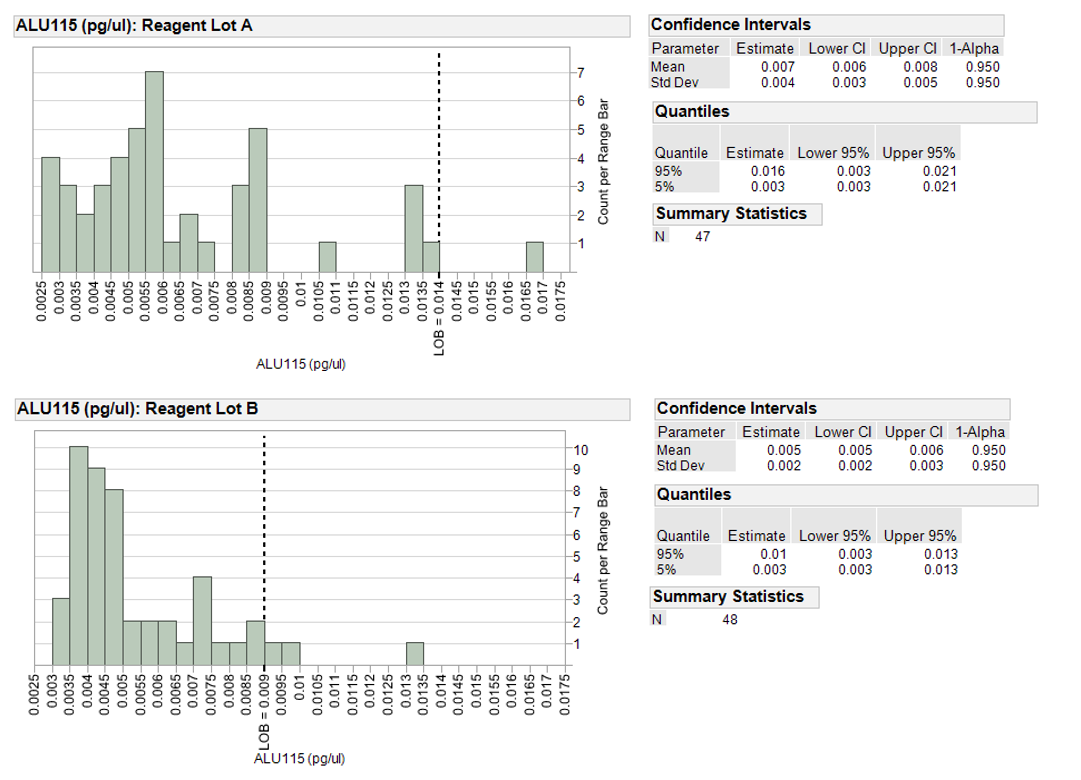

Supplement: S2 Fig — LoB, calculated according to the CLSI nonparametric option [91] and denoted by the dashed vertical line is 0.014 pg/μl. (TIF) [file pone.0227385.s002.tif]

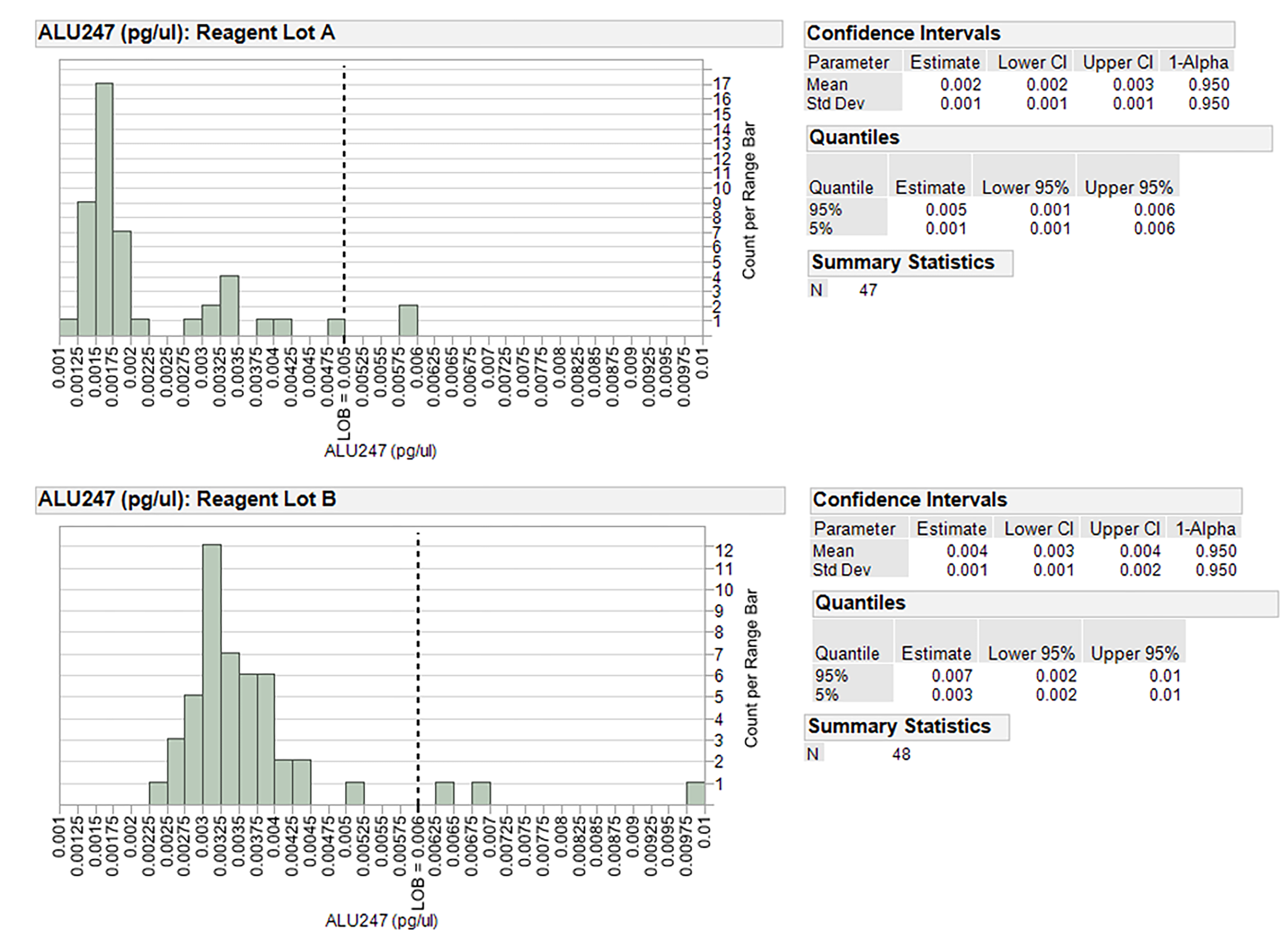

Supplement: S3 Fig — LoB, calculated according to the CLSI nonparametric option [91] and denoted by the dashed vertical line is 0.006 pg/μl. (TIF) [file pone.0227385.s003.tif]

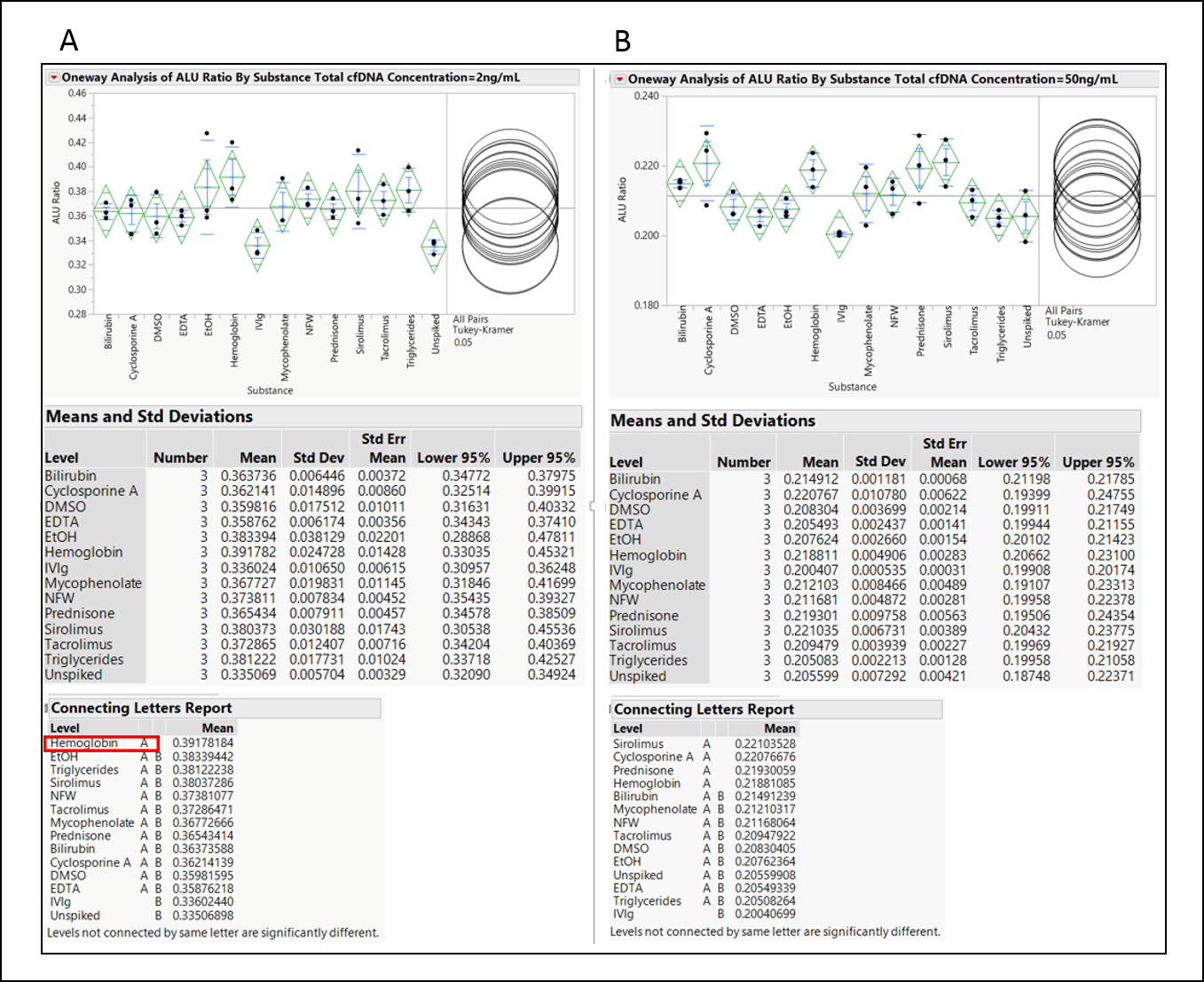

Supplement: S4 Fig — Testing results at 2ng/ml TCF (A) and at 50 ng/ml TCF (B). For results at 25 ng/ml TCF see Text, Fig 7. (TIF) [file pone.0227385.s004.tif]

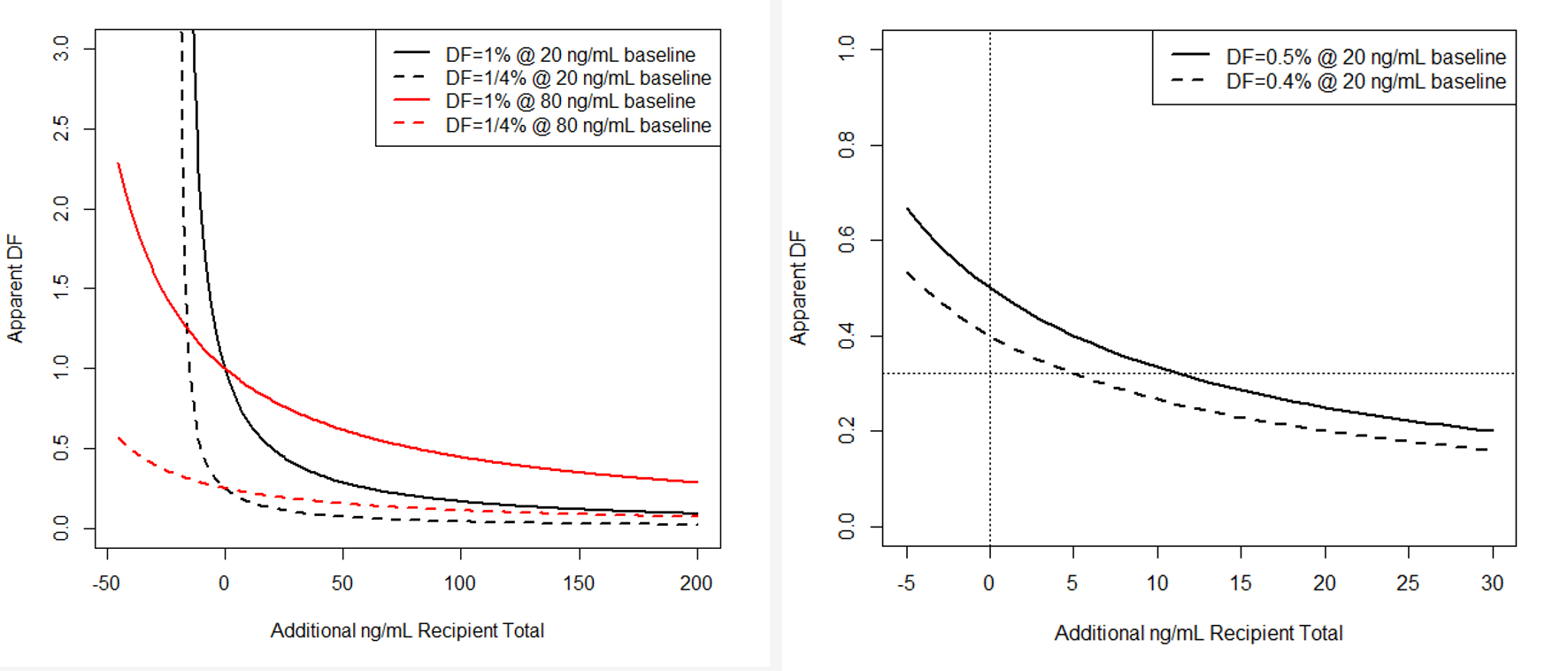

Supplement: S5 Fig — (TIF) [file pone.0227385.s005.tif]

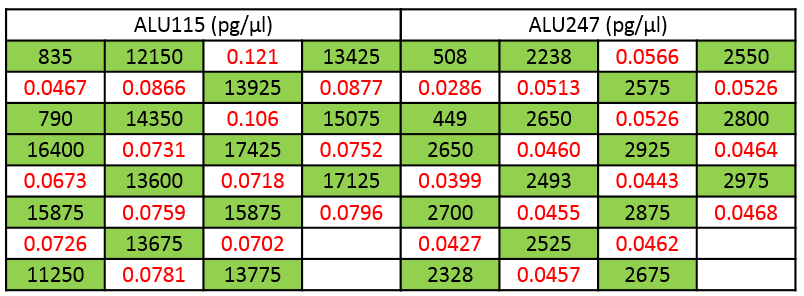

Supplement: S6 Fig — (TIF) [file pone.0227385.s006.tif]

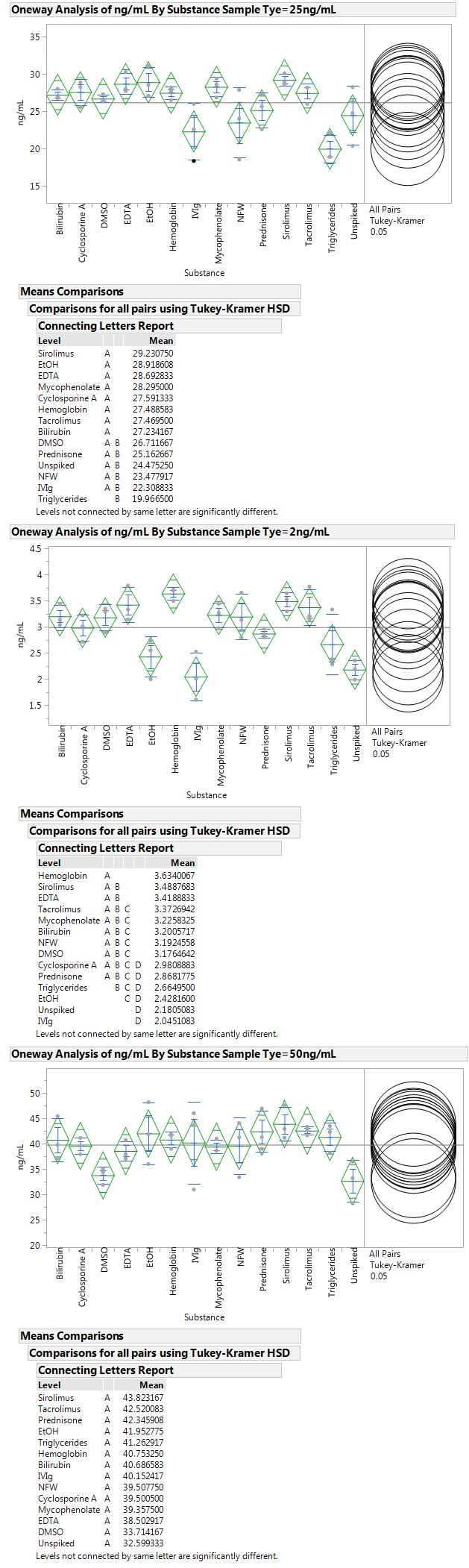

Supplement: S7 Fig — (TIF) [file pone.0227385.s007.tif]

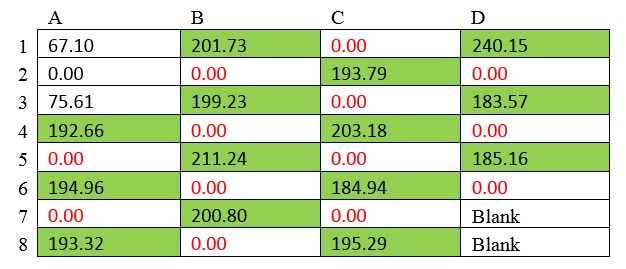

Supplement: S8 Fig — (TIF) [file pone.0227385.s008.tif]

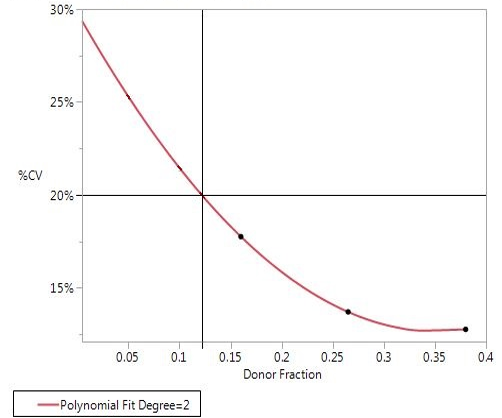

Supplement: S9 Fig — The average %CV at each DF is shown, along with the calculated best-fit line. The DF level along that line at which precision of the assay was ≤ 20%CV, is 0.108%, indicated by the vertical marker. (TIF) [file pone.0227385.s009.tif]

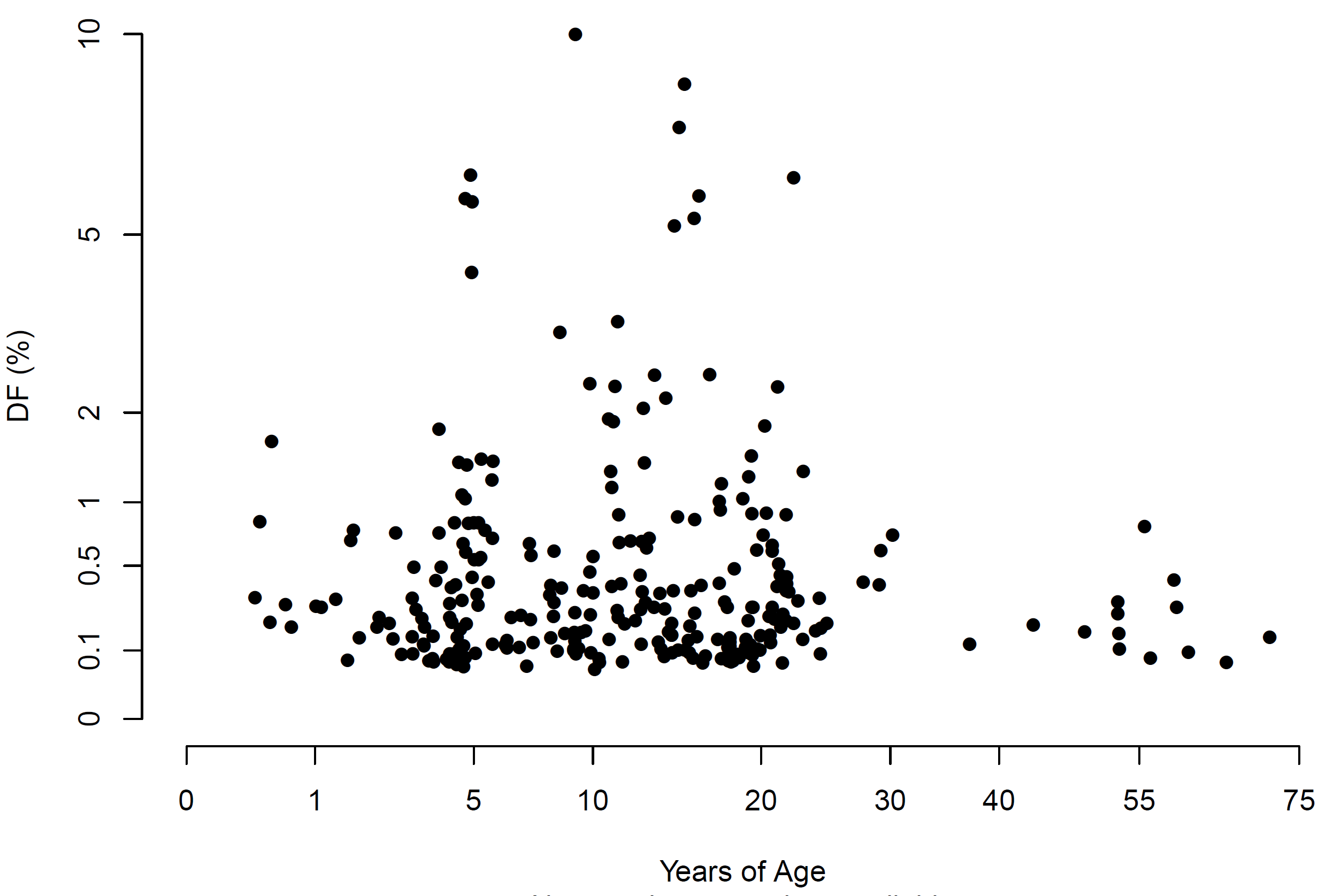

Supplement: S10 Fig — Data includes study subjects (N = 180) and clinical registry records (N = 96). See text for statistical interpretation. (TIF) [file pone.0227385.s010.tif]
