# Supplementary material for: Cell-free DNA donor fraction analysis in pediatric and adult heart transplant patients by multiplexed allele-specific quantitative PCR: Validation of a rapid and highly sensitive clinical test for stratification of rejection probability
Source: PLoS One. 2020 Jan 13;15(1):e0227385. doi: 10.1371/journal.pone.0227385 (PMC6957190; doi:10.1371/journal.pone.0227385)
Supplement: S1 Table — (DOCX) [file pone.0227385.s011.docx]

| **Interfering Substance** | **Form** | **Testing Concentration** | **Solvent** | **CLSI Recommendation** |
| --- | --- | --- | --- | --- |
| Bilirubin Conjugate | Lyophilized powder | 0.2mg/ml | in Water | 20mg/dl |
| Hemoglobin | Lyophilized powder | 5mg/ml | in water | 500mg/dl |
| EDTA | Powder (anhydrous) | 0.001mg/ml | in Water | 3.4umol/L |
| Prednisone | 1g in poly tube | 0.0003mg/ml | in ethanol | 0.84umol/L |
| FK-506 monohydrate (Tacrolimus) | Powder | 40.2ng/ml | in DMSO | 50nmol/L |
| Rapamycin (sirolimus) | neat | 12ng/ml | in DMSO | Upper therapeutic Range |
| Mycophenolate (mycophenolate mofetil) | Powder | 3.5mcg/ml | in DMSO | Upper therapeutic Range |
| Cyclosporine A | powder | 400ng/ml | in DMSO | Upper therapeutic Range |
| Triglycerides | 20% Emulsion | 30mg/ml | N/A | 3000mg/dl |
| Gammaguard (IVIg) | 10% Solution | 11mg/ml | N/A | Upper therapeutic Range |
| CMV Virus | Solution | 10,000 copies/ml | N/A | High viral load associated with tissue damage (Mayo Clinic) |
| BKV Virus | Solution | 10,000 copies/ml | N/A | High viral load associated with tissue damage (Mayo Clinic) |

**S1 Table. Interfering Substance Testing Concentrations**
